# Determining Quasi-Equilibrium Electron and Hole Distributions of Plasmonic Photocatalysts Using Photomodulated X-ray Absorption Spectroscopy

**DOI:** 10.1021/acsnano.3c08181

**Published:** 2024-03-18

**Authors:** Levi Daniel Palmer, Wonseok Lee, Chung-Li Dong, Ru-Shi Liu, Nianqiang Wu, Scott Kevin Cushing

**Affiliations:** 1 Division of Chemistry and Chemical Engineering, California Institute of Technology, Pasadena 91125, California, United States; 2 Department of Physics, Tamkang University, New Taipei City 251301, Taiwan; 3 Department of Chemistry, National Taiwan University and Advanced Research Center for Green Materials Science and Technology, Taipei 10617, Taiwan; 4 Department of Chemical Engineering, University of Massachusetts Amherst, Amherst 01003−9303, Massachusetts, United States

**Keywords:** X-ray absorption, X-ray spectroscopy, Bethe-Salpeter equation, plasmonics, core−shell nanoparticles, hot carriers, photocatalysis

## Abstract

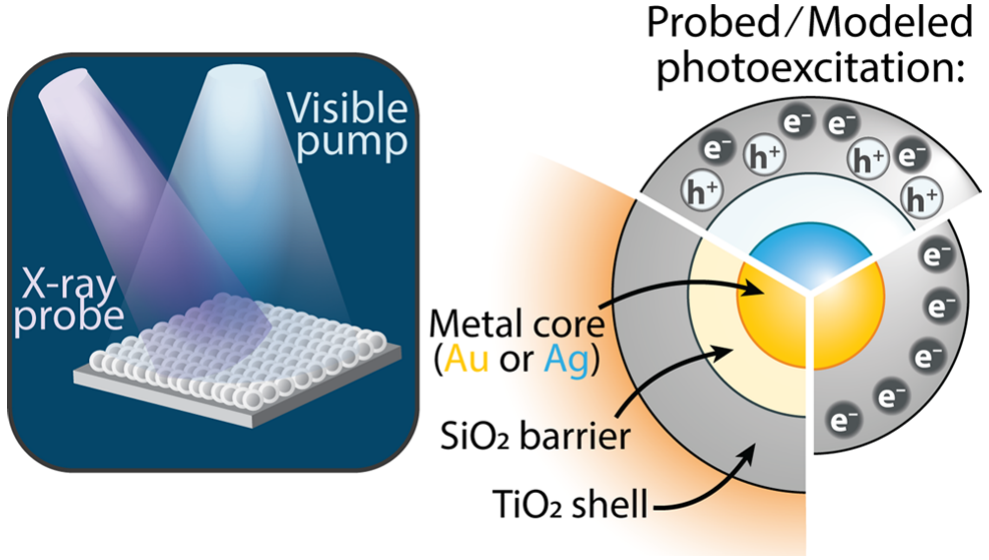

Most photocatalytic and photovoltaic devices operate under broadband, constant illumination. Electron and hole dynamics in these devices, however, are usually measured by using ultrafast pulsed lasers in a narrow wavelength range. In this work, we use excited-state X-ray theory originally developed for transient X-ray experiments to study steady-state photomodulated X-ray spectra. We use this method to attempt to extract electron and hole distributions from spectra collected at a nontime-resolved synchrotron beamline. A set of plasmonic metal core–shell nanoparticles is designed as the control experiment because they can systematically isolate photothermal, hot electron, and thermalized electron–hole pairs in a TiO_2_ shell. Steady-state changes in the Ti L_2,3_ edge are measured with and without continuous-wave illumination of the nanoparticle’s localized surface plasmon resonance. The results suggest that within error the quasi-equilibrium carrier distribution can be determined even from relatively noisy data with mixed excited-state phenomena. Just as importantly, the theoretical analysis of noisy data is used to provide guidelines for the beamline development of photomodulated steady-state spectroscopy.

A balance between carrier photoexcitation, thermalization, and recombination rates determines the quasi-equilibrium carrier distribution that controls photocatalytic and photovoltaic device efficiencies (Figure 1).^1−3^ A quasi-equilibrium state occurs during the thermodynamic balance of the system’s photoexcitation and relaxation. Photoexcited carriers are generally assumed to be fully thermalized to the band edges at the device’s working conditions.^4^ However, slowed hot carrier cooling through phonon bottlenecks,^5^ surface-state trapping,^6−9^ or dielectric carrier Coulomb screening^10^ can generate a nonthermal carrier quasi-equilibrium. In nanoscale junctions, photoexcited carriers can transfer between active layers or to surface catalysts on timescales shorter than carrier thermalization.^11,12^ Transferring the quasi-equilibrium hot carrier population into surface reactants then modifies a semiconductor’s photochemical redox potential, tailoring resultant reaction products.^13^ For example, plasmonic metal–semiconductor junctions have been used to increase semiconductors’ photocatalytic product selectivity^14−16^ and solar power conversion efficiency.^17,18^

**Figure 1:**
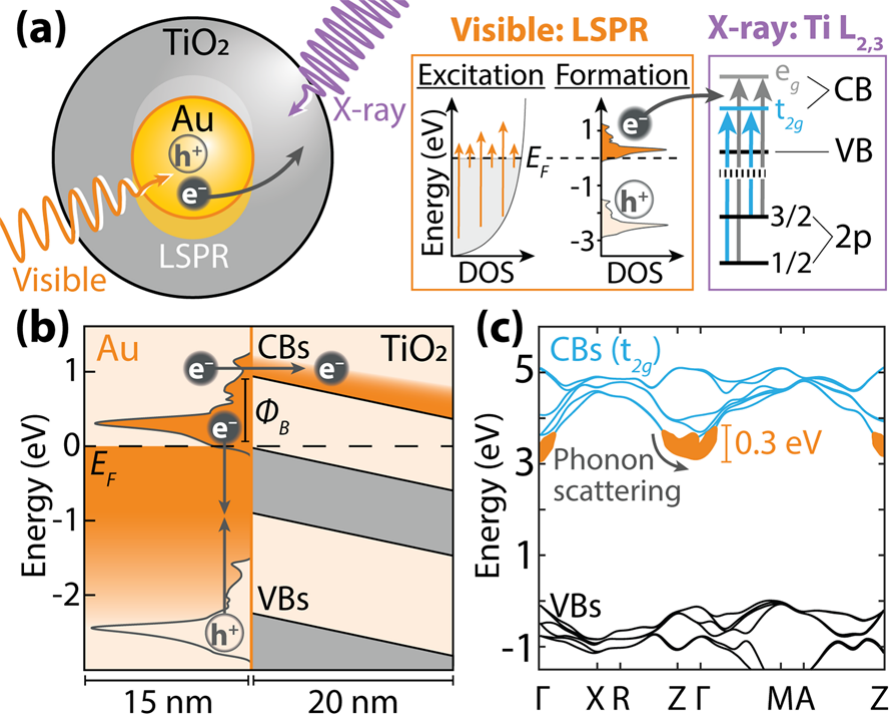
**Photoexcited properties of plasmonic core–shell nanoparticles.** (a) Continuous photoexcitation of a metal nanoparticle’s LSPR with visible light results in dynamic carrier excitation [ref (2)]. Hot carrier formation then occurs following electron–electron and electron–phonon scattering [ref (3)]. The hot carriers transfer into TiO_2_ and can be probed with the Ti L_2,3_ edge. (b) The hot carriers transfer over the Au@TiO_2_ Schottky barrier (ϕ_B_) and fill the TiO_2_ CBs with an excess energy of 0.3 eV and subsequently (c) thermalize in the CBs through phonon scattering.

Measuring the quasi-equilibrium carrier distribution is therefore critical. However, few methods to date can characterize the equilibrium photoexcited carrier population with the same detail as ultrafast pump–probe methods like two-dimensional, terahertz, or photoemission spectroscopies.^19−22^ While ultrafast spectroscopy is the conventional method for measuring carrier thermalization and recombination, ultrafast measurements sum over different relaxation pathways, often use a high peak power that exceeds the solar flux, and rely on laser sources that are more narrowband than the solar spectrum. When effects such as Fermi-level pinning, defects, and surface states are present, it can be difficult to reconstruct steady-state carrier distributions by using ultrafast measurements alone.

X-ray spectroscopy is one potential method for resolving carrier distributions and dynamics. Transient X-ray spectroscopy is now routinely used to measure element-specific electron and hole energies in multielement catalysts.^23−26^ The same capabilities should also be true for steady-state, photomodulated X-ray spectroscopy.^27^ However, measuring and interpreting photomodulated X-ray spectra are challenging tasks because the decreased photoinduced carrier concentration and slower repetition rate make the signal-to-noise ratio (SNR) significantly lower. Therefore, accurate excited-state X-ray theory is needed, even more so than ultrafast X-ray spectroscopy, to interpret the small photomodulated spectral intensity within the experimental noise.

Previous investigations of plasmonic Au@TiO_2_ nanoparticles and their photomodulated X-ray spectra suggest that quasi-equilibrium hot electron populations exist in TiO_2_.^7,28,29^ In our prior work, the photoexcited X-ray spectra were not modeled fully *ab initio* but were rather calculated using a semiquantitative model of phase-space filling and lifetime effects.^29^ Thermal and photoexcited hole effects were not included. Here, we use excited-state density functional theory (DFT) and Bethe-Salpeter equation (BSE) calculations to evaluate steady-state photomodulated X-ray spectra. To simulate hot electron X-ray spectra, we fill the DFT-calculated conduction band states with electrons from the conduction band minimum (CBM) to a specified energy and calculate the corresponding spectrum with the BSE. Methods that model transient charge configurations *ab initio* instead use density functional perturbation theory and the Boltzmann transport equation to predict ultrafast charge redistribution following electron–phonon interactions and transport.^30^ However, density functional perturbation theory is not used for modeling steady-state dynamics due to computational costs.

In this work, we tested whether photomodulated, steady-state X-ray spectroscopy can be used to quantify quasi-equilibrium carrier distributions. X-ray absorption at the Ti L_2,3_ edge is measured for each nanoparticle with modulated photoexcitation. An adiabatic approximation of the BSE is then used to predict the change in the X-ray spectrum for each possible photoexcited configuration. We use a mean squared error (MSE) analysis to compare the potential theoretical contributions of thermal, hot electron, and dipolar excitations to the signal from plasmonic core–shell nanoparticles designed to isolate each one of these effects. Even in the case of relatively noisy spectra, quasi-equilibrium hot carrier distributions are differentiated from photothermal heat. Separating electron from hole effects on a photothermal background is more difficult because of the hole’s smaller perturbation to the Ti L_2,3_ edge. Within experimental noise, electron versus hole populations were not separable by statistical significance. However, our calculations demonstrate that electrons and holes have distinct spectral features and could be differentiated with improved X-ray measurements, and we discuss the required experimental conditions for such measurements. Our findings suggest that photomodulated X-ray spectroscopy at nontime-resolved beamlines can be used to separate electron, hole, and thermal excitations, but continued improvement in experimental SNRs is needed when multiple photoexcited processes simultaneously exist.

## Results and Discussion

**Figure 2:**
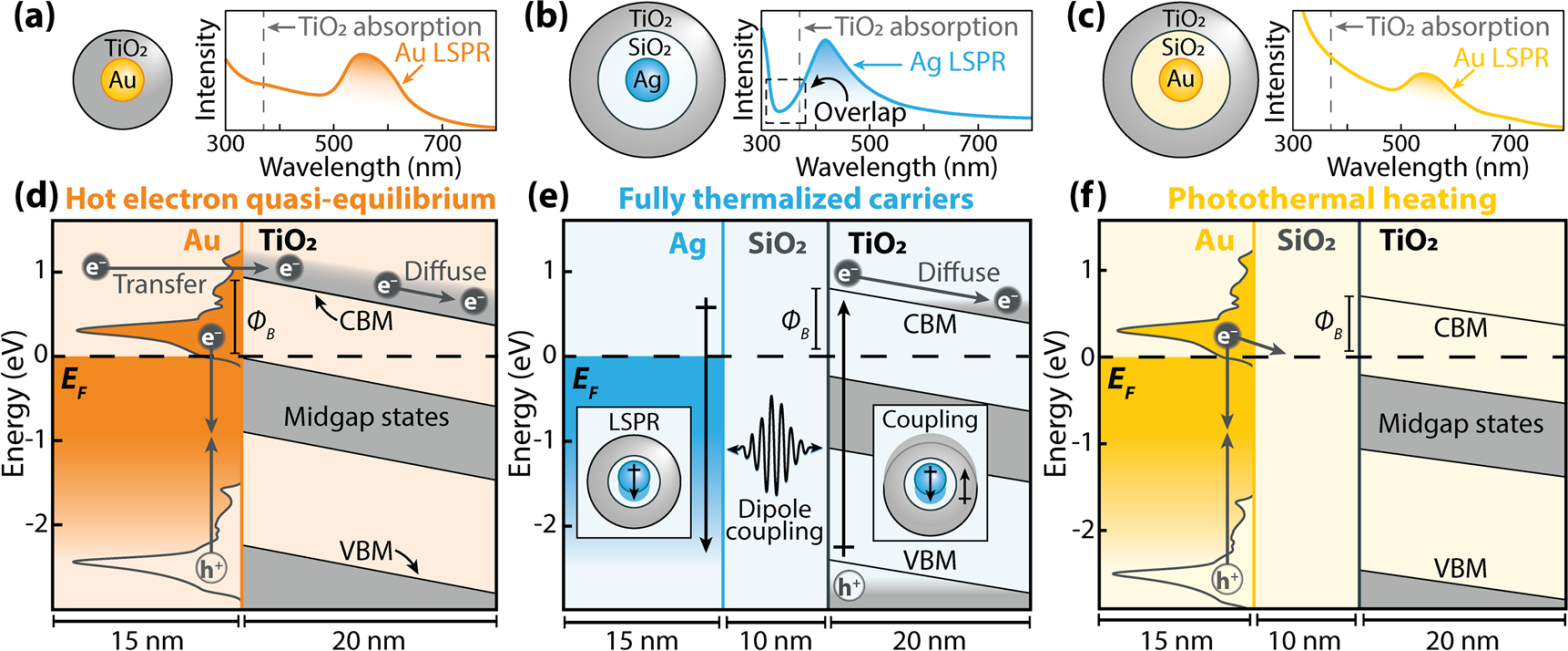
**Plasmonic core–shell nanoparticle heterostructure characterization.** (a–c) UV–visible absorption spectra for (a) Au@TiO_2_, (b) Ag@SiO_2_@TiO_2_, and (c) Au@SiO_2_@TiO_2_ core–shell nanoparticles. The LSPR and UV bandgap for amorphous TiO_2_ (gray dashed line at 3.34 eV, 370 nm) are marked [refs (43, 44)]. (d–f) Schematic representations of each nanoparticle’s band alignment and hot carrier distribution. (d) Hot electrons up to ∼0.3 eV above the CBM, relative to the Schottky barrier (ϕ_B_), have sufficient energy to transfer into TiO_2_ directly [ref (3)]. (e) The Schottky barrier and SiO_2_ layer prevent hot electron transfer. Instead, the localized electromagnetic field from the plasmon couples with TiO_2_ electron–hole excitation, and carriers are created at both the CBM and the VBM. (f) The SiO_2_ layer prevents electron transfer into TiO_2_.

A nanoparticle’s localized surface plasmon resonance (LSPR) can be used to transfer energy to a semiconductor through multiple mechanisms. Here, a SiO_2_ layer is used to systematically control three such mechanisms between a Au or Ag nanoparticle core and a TiO_2_ shell. For Au@TiO_2_ nanoparticles, plasmonic hot electrons in Au can overcome the interfacial Schottky junction to inject into TiO_2_ (Figure 2a,d).^3,31,32^ Ag@SiO_2_@TiO_2_ nanoparticles use the plasmon’s dipole moment to increase the light absorption rate in the tail of a semiconductor’s absorption edge, creating electron–hole pairs (Figure 2b,e).^20,28^ In Au@SiO_2_@TiO_2_ nanoparticles, a SiO_2_ layer prevents carrier transfer from Au into TiO_2_, so the TiO_2_ shell only experiences heating from the Au core to provide a control experiment (Figure 2c,f).^28^ Past work has verified that these core–shell nanoparticles isolate these excited-state effects.^28^

This paper theoretically interprets previously measured X-ray spectra of the nanoparticles synthesized and characterized in ref (28). In these previous measurements, the nanoparticles were reported to have a 15 nm radius Au or Ag core, a 10 nm SiO_2_ insulating layer (not present in Au@TiO_2_), and a 10–20 nm amorphous TiO_2_ outer shell. UV–visible absorption spectroscopy was used to measure the LSPR center wavelength at 420 nm for Ag and 560 nm for Au (Figure 2a–c).

The approximate interfacial band bending of each heterojunction is calculated using a 1D drift-diffusion model implemented in the Automat FOR Simulation of HETerostructures (AFORS-HET) (Figure 2d–f).^33^ This approach does not consider nanoscale near-field or photoexcited effects. The approximate Schottky barriers are 0.9 eV for Au@TiO_2_, 0.8 eV for Ag@SiO_2_@TiO_2_, and 0.7 eV for Au@SiO_2_@TiO_2_. The metal–semiconductor junction produces band bending and built-in electric fields in the TiO_2_ and SiO_2_ layers. The average built-in field estimated for the semiconducting layers is ∼10^5^ V/cm. The SiO_2_ insulator acts as a carrier tunnelling barrier between the metal and TiO_2_, and the Schottky barrier in such cases refers to the energetic barrier for electron transfer at the SiO_2_–TiO_2_ interface. Considering the ∼4.4 eV Au-SiO_2_ Schottky barrier, which exceeds the maximum hot electron energy by 3.2 eV, Au hot carriers would need to tunnel through SiO_2_ to reach TiO_2_. Similar junctions with a 4.8 nm SiO_2_ oxide were previously measured to have a <10^–10^ A/cm^2^ tunneling current at a 10^5^ V/cm applied bias.^34^ Therefore, as experimentally observed, photoexcited electrons would not transfer to TiO_2_ for the Au@SiO_2_@TiO_2_ system. See the Supporting Information for numerical input parameters and field calculation.

**Figure 3:**
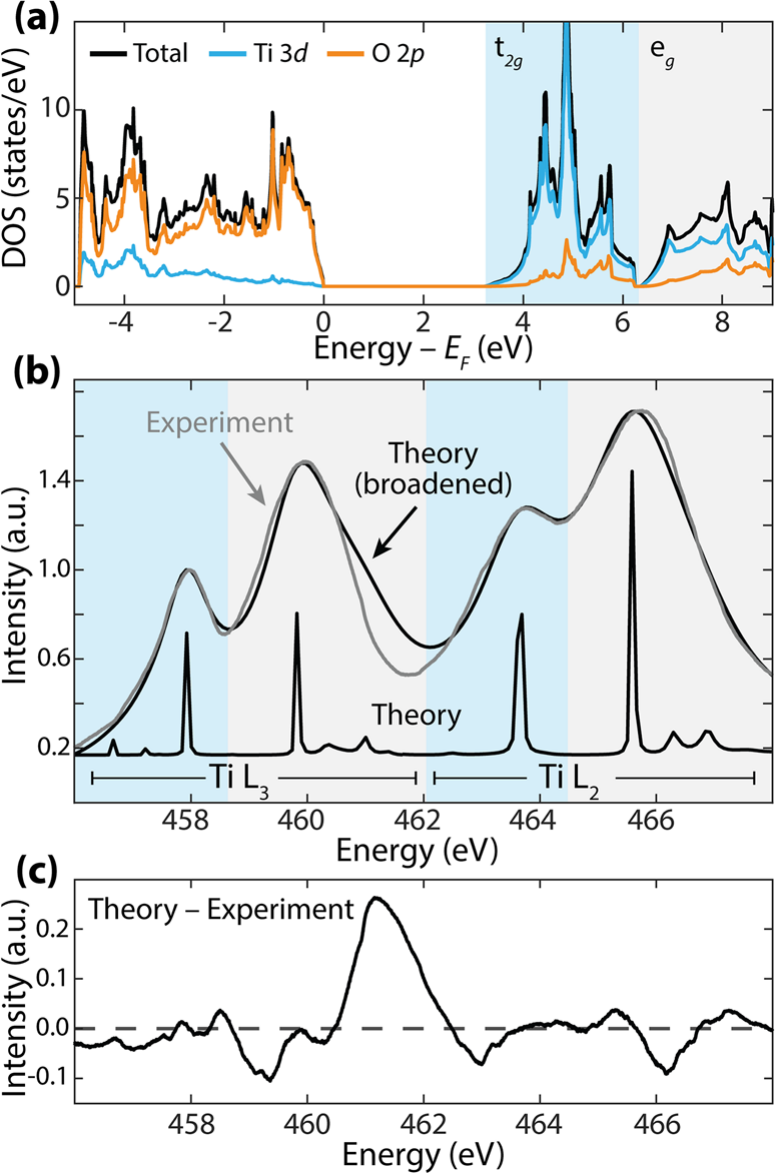
**Calculation of TiO**_**2**_**electronic structure and X-ray absorption.** (a) DFT-calculated, projected density of states for anatase TiO_2_. The Fermi level (E*_F_*) represents the valence band edge. (b) Calculated (black) and measured (gray) Ti L_2,3_ X-ray spectra for TiO_2_. The theory spectrum is broadened to match the experiment with the bottom spectrum being the unbroadened output. The blue and gray shading depict the Ti t_2g_ and e_g_ states, respectively. (c) The difference between theory and experiment.

The ground-state electronic structure and X-ray absorption of TiO_2_ are first calculated as shown in Figure 3. The Ti L_2,3_ X-ray absorption edge (456–468 eV) was measured, which corresponds to a core electron transition from Ti 2p to Ti 3d states (Figure 1a, right). The DFT calculated projected density of states (PDOS) for anatase TiO_2_ is given in Figure 3a. A 1 eV scissor shift is applied to the bandgap. In the PDOS, the O 2p orbitals dominate the valence band and the Ti 3d orbitals compose the conduction band. The crystal field characteristically splits the Ti 3d conduction band into the t_2g_ (blue shading) and e_g_ (gray shading) orbitals in the electronic structure and ground-state X-ray absorption (Figure 3a,b).^35^

Figure 3b compares the BSE simulated Ti L_2,3_ edge to the measured ground-state Au@SiO_2_@TiO_2_ spectrum. An energy-dependent broadening method of the predicted spectrum was used to replicate the experimental core-hole lifetime broadening, which has a 3:2 broadening ratio (L_2_:L_3_) for the TiO_2_ Ti L_2,3_ edge (see Supporting Information).^36^ According to the PDOS in Figure 3a, the photomodulated Ti L_2,3_ edge predominantly probes photoexcited electrons over holes through the Ti 3d states; however, because of the screening and angular momentum coupling matrix elements in the X-ray transition Hamiltonian, holes will still perturb the core-to-valence transition excitons.^37,38^

This work approximates the nanoparticle’s amorphous TiO_2_ as purely anatase phase. Although 10–20 nm TiO_2_ nanoparticles typically consist of a mixture of anatase and brookite, anatase is a slightly more stable phase, and this approximation reduces the otherwise insurmountable computational costs of excited-state X-ray BSE calculations for hundreds of atoms.^39−42^ We find this to be a valid approximation due to the excellent match between the ground-state experiment and theory (Figure 3b). However, aspects of the amorphous phase electronic structure are not considered. First, there is a discrepancy between anatase and amorphous TiO_2_ for the Ti L_3_ e_g_ states at 461 eV (Figure 3c).^43^ The core-hole exciton effects calculated by BSE are therefore not accurately modeled at these energies and are not considered during the MSE analysis. Further, defect-induced midgap states (depicted in Figure 2d–f) are not modeled. Midgap states show little effect on the ground-state TiO_2_ spectra but may appear as a shoulder of the L_2_ t_2g_ peak at 463 eV (Figure 3b,c).

**Figure 4:**
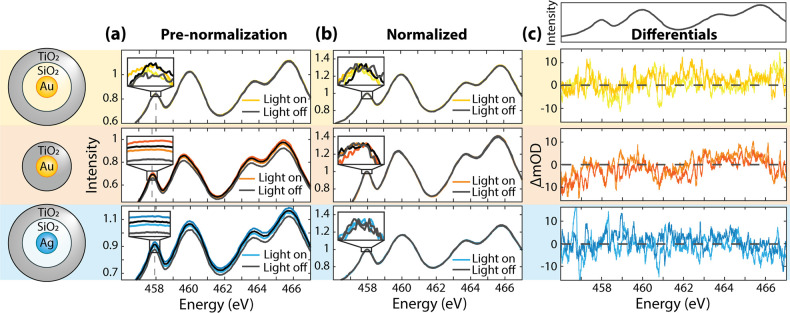
**X-ray absorption spectra of core–shell nanoparticles with and without photoexcitation.** (a) Raw light on and light off Ti L_2,3_ edge X-ray spectra of the amorphous TiO_2_ outer shell. The spectral intensity increased throughout the data collection of all four spectra. The spectra are background-subtracted. Each inset magnifies the edge maxima’s intensity differences caused by charging. Each inset window size is 0.15 eV width but a variable amplitude. (b) Spectra from (a) normalized to the edge maximum near 458 eV (gray dashed line) to correct for charging that broadly increases the spectral amplitude. (c) Photoexcited differential spectra of the normalized data in (b) to highlight photomodulated energetic shifts in the L_2,3_ edge. The lighter spectra are from the first light on, light off collection. The ground-state experimental spectrum is shown above for reference.

The previously measured X-ray spectra were collected using a light-on, light-off sequence with a 1 min collection time per spectrum. A continuous-wave lamp, filtered below the 3.34 eV amorphous TiO_2_ bandgap, was used to photoexcite the nanoparticles’ LSPR.^44,45^ Surface charging from photoexcitation creates a baseline drift in photomodulated spectra for the total electron yield detection method.^46^ To account for charging, each spectrum is normalized to the edge onset maximum near 458 eV (gray dashed line in Figure 4) after the baseline background subtraction. The charging normalization creates artifacts directly below and above the X-ray absorption edge, so only the 458–466 eV range is compared to theory herein (see average spectra for all samples overlaid in Figure S5). Charging is not measured for the Au@SiO_2_@TiO_2_ sample, which confirms that only heat results from photoexcitation. However, the insets of Figure 4a reflect that even the light-off spectra change after modulating photoexcitation, meaning residual charges may perturb the total electron yield acquisition even after sample relaxation.

The measured differential absorption, calculated as the log of spectra collected with the lamp on divided by those with the lamp off and averaged across two data sets, is used to identify photoexcited carrier and structural effects on the X-ray spectra (Figure 4). Because the total electron yield detection only probes the first ∼4 nm of TiO_2_, only carriers in surface trap states in the amorphous TiO_2_ are probabilistically measured.^47^ The spectra in Figure 4 are relatively noisy due to the lower power of the excitation source and slower modulation time of the steady-state measurement. Therefore, we first test the accuracy of our *ab initio* approach by comparing to a previous ultrafast X-ray absorption spectrum of anatase TiO_2_ (Figure S1).^27^ The measured transient spectrum is analyzed using an adiabatic approximation to excited-state effects in the BSE. This approach has been verified previously for other transient X-ray data sets and is described in the Methods section.^26,37,38^ The ultrafast time slice is after carrier thermalization (1 ps after photoexcitation). The proposed *ab initio* method accurately reproduces the transient X-ray spectrum at all energies besides 458–460 eV. The discrepancy is likely due to the reported onset of carrier transfer to midgap states (see Supporting Information). The electron, hole, and thermal signals are reproduced in this case, giving a baseline for the accuracy of the photomodulated data presented here.

Given this verification, we proceed to analyze the photomodulated spectra. Three differences are observed between each plasmonic excitation mechanism, although it must be noted that, since only two averages are used, the statistical significance must be interpreted with caution. First, all nanoparticle’s spectra have different amplitudes just after the L_3_ edge at 458 eV. Second, the Au@TiO_2_ has decreased absorption centered at 462 eV. Lastly, the Ag@SiO_2_@TiO_2_ nanoparticles have decreased absorption at 465.5 eV. We then investigate how these trends compare to the theoretically predicted photomodulated spectra, which is found to successfully model ultrafast dynamics (Figure S1).

**Figure 5:**
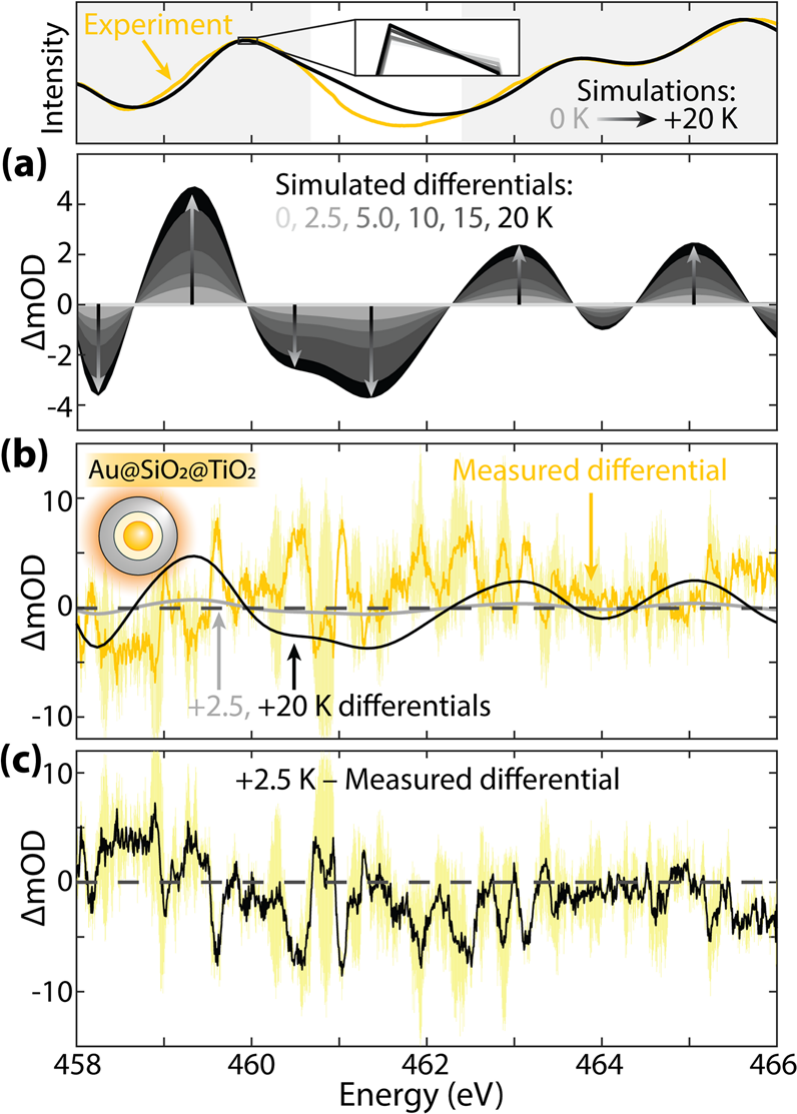
**Photothermal effects on the Ti L**_**2,3**_**edge in Au@SiO**_**2**_**@TiO**_**2**_**.** (Top) Raw spectra for reference. The spectral range analyzed by the MSE is shaded. (a) Simulated differential X-ray spectra for a 0–20 K anatase lattice expansion. The arrows highlight the differential amplitude change with an increasing lattice parameter. (b) The lattice-expanded differential spectra predicted for 2.5 and 20 K heating are compared to the Au@SiO_2_@TiO_2_ differential spectrum. The yellow shaded region depicts the experiment’s standard deviation. (c) The measured differential subtracted from the optimized simulation differential at 2.5 K, selected using the MSE analysis. The experiment’s standard deviation (yellow shading) is included for reference.

The steady-state spectral signatures of photothermal effects are first compared to theory by using the Au@SiO_2_@TiO_2_ experimental control (Figure 5). Photothermal heating arises from the heat produced by carrier thermalization in the metal nanoparticle after LSPR photoexcitation and relaxation.^48^ The photomodulated Au@SiO_2_@TiO_2_ nanoparticles’ experimental spectra lack the surface charging artifact that results from photoexcited carriers in the other two nanoparticle systems (Figure 4a), confirming that photoexcited carriers are not excited in TiO_2_.

Heating is modeled through DFT and BSE calculations by an expansion of the TiO_2_ lattice. Calculations are performed for lattice expansions equivalent to 2.5, 5.0, 10, 15, and 20 K temperature increases above 300 K (Figure 5a).^49^ The spectrum’s peak positions linearly red-shift with increasing lattice temperature, increasing and decreasing the differential intensity at pre- and postedge regions, respectively. The spectrum mainly red-shifts because of the Ti atoms’ reduced crystal field. The simulated X-ray differential absorption for a 2.5 K lattice-expanded TiO_2_ crystal is compared to the measured differential absorption in Figure 5b.

The Au@SiO_2_@TiO_2_ nanoparticle temperature after photoexcitation was predicted to be +2.5 K through a MSE fit of all simulations in Figure S2. Subtracting the 2.5 K heating differential from the experiment (Figure 5c) reflects that the general spectral changes are reproduced. The heating simulation’s largest disagreement results from the anatase TiO_2_ approximation at 460–462 eV. The differential between theory and experiment shows bias, as in it is not centered around zero, so a statistical conclusion is difficult even if trends are similar by eye. The MSE fit predicts an ∼2.5 K rise in the TiO_2_ layer and is consistent, within an order of magnitude, with other studies reports of 7.7 K (theoretical)^50^ and 2.6 ± 2.3 K (experimental)^51^ heating of aqueous Au nanoparticles when using similar excitation densities. The thermal dissipation will, of course, differ in the vacuum environment for the experimental X-ray measurements.

**Figure 6:**
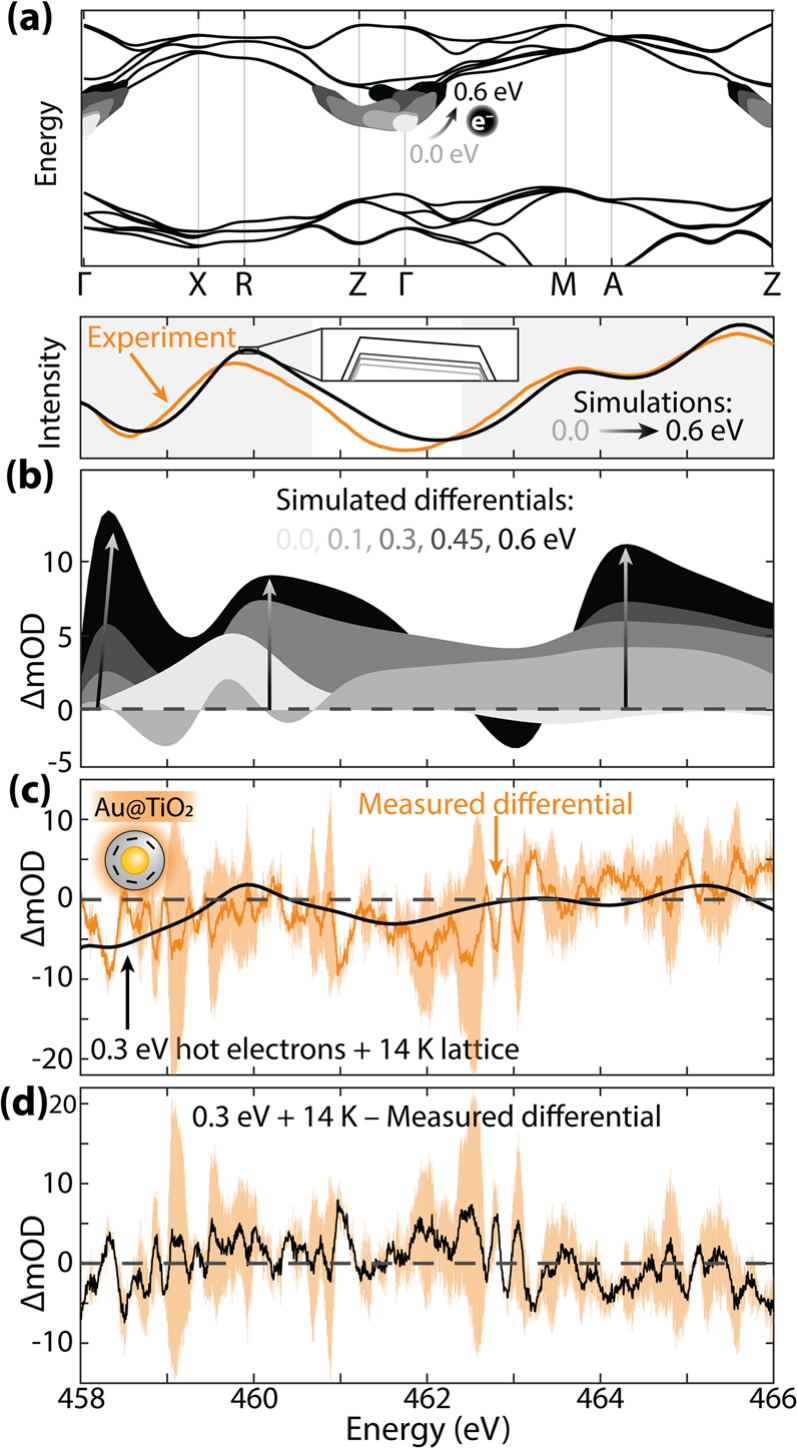
**Hot electron effects on the Ti L**_**2,3**_**edge in Au@TiO**_**2**_**.** (a) Simulated hot electrons 0.0, 0.1, 0.3, 0.45, and 0.6 eV above the TiO_2_ CBM. (b, Top) Raw spectra for reference. The spectral range analyzed by the MSE is shaded. (b) Simulated differential X-ray spectra for each hot electron occupation in TiO_2_. The spectral intensity is normalized to the number of electrons simulated in all but the thermalized (0.0 eV) case. (c) The optimized simulation with hot electrons 0.3 eV above the CBM with a 14 K lattice expansion compared to the Au@TiO_2_ measured differential absorption. The orange shaded region depicts the experiment’s standard deviation. (d) The measured differential subtracted from the optimized simulation differential shown in (c) with the standard deviation.

Next, the Au@TiO_2_ nanoparticle sample that has both photothermal and hot electron effects is examined. To simulate hot electron transfer in the Au@TiO_2_ nanoparticles, electrons up to a specific energy above the TiO_2_ CBM (0.0 eV for fully thermalized electrons, 0.1, 0.3, 0.45, and 0.6 eV) are included in the BSE calculation (Figure 6a). We simulate spectra by approximating an average electron energy in the CBs and not a distribution. The added electrons change the core-hole screening and prevent X-ray transitions into the newly blocked states, leading to complex differential absorption, as shown in Figure 6b. In Figure 6b, the intensity of the hot electrons’ differential absorption is normalized to the total number of simulated hot electrons to better evaluate excited-state trends with increasing hot electron energy.

Unlike photothermal heating, changes in TiO_2_’s simulated differential absorption are not perfectly linear with increasing electron energy (Figure 6b). Instead, spectral intensity increases with the hot electron energy, and there are differential peaks from changes in the screening of the core–valence exciton and X-ray transitions blocked by hot electrons. The differential peaks are mainly a result of hot electrons affecting the screening and angular momentum components of the core–valence exciton when measured energies are above the hot carriers at the bottom of the t_2g_ bands (Figure 6a). The differential features blue-shift as more hot electrons screen the exciton in the BSE. State-filling effects of hot electrons blocking X-ray transitions begin to appear at 463 eV when the simulated hot electrons fully occupy states above 0.45 eV.

The measured Au@TiO_2_ nanoparticle differential absorption spectrum is compared to a simulated differential spectrum with 0.3 eV hot electrons and 14 K lattice expansion in Figure 6c. Compared to Au@SiO_2_@TiO_2_, the simulated X-ray spectrum with hot electrons has a new minimum around 462 eV, consistent with the measured spectral differences between samples with and without hot electrons (Figure 4). An MSE analysis was used to determine the most likely temperature and hot electron energy based on the simulated spectra. Each modeled hot electron distribution is shown separately in Figure S9, and the differential X-ray spectra with both hot electrons and temperature simulated are in Figure S11. Here, the differential between the experiment and theory is centered around zero, but again, the experimental results must be interpreted with some caution given the low number of averages. Using the MSE fit, the TiO_2_ lattice temperature is found to be hotter than for the Au@SiO_2_@TiO_2_ nanoparticles, attributed to the larger plasmon intensity that leads to a greater level of hot electron thermalization in TiO_2_ (Figure S3). Further, the 0.3 eV hot electron quasi-equilibrium distribution is consistent with recent studies, as steady-state and ultrafast Raman measurements estimate that hot electrons exceeding 0.32 and 0.34 eV, respectively, transfer from Au nanoparticles to nearby molecules.^52,53^ An approximate calculation comparing the electron excitation and relaxation rates in the amorphous TiO_2_ is given in the Supporting Information, but the main conclusions of Figure 6 are that photothermal and hot electron effects can be differentiated within a relatively noisy spectrum.

Further light-intensity-dependent control experiments would be necessary to quantify the hot electron concentration and would also be useful to clarify the nonlinear change with hot carrier concentration versus the temperature-induced shift. We approximate the measured hot electron density using the relative occupation of the state filling in the band structure. The state-filling simulation for states up to 0.3 eV above the CBM best matches the experiment (Figures 6c and S2b). The integrated total DOS in this energy range is 150 states of the 10^4^ total possible calculated conduction states. Using this number, and the measured DOS for nanocrystalline TiO_2_ (8 × 10^18^ cm^–3^), one can approximate an electron concentration of ∼10^16^ cm^–3^.^54^

**Figure 7:**
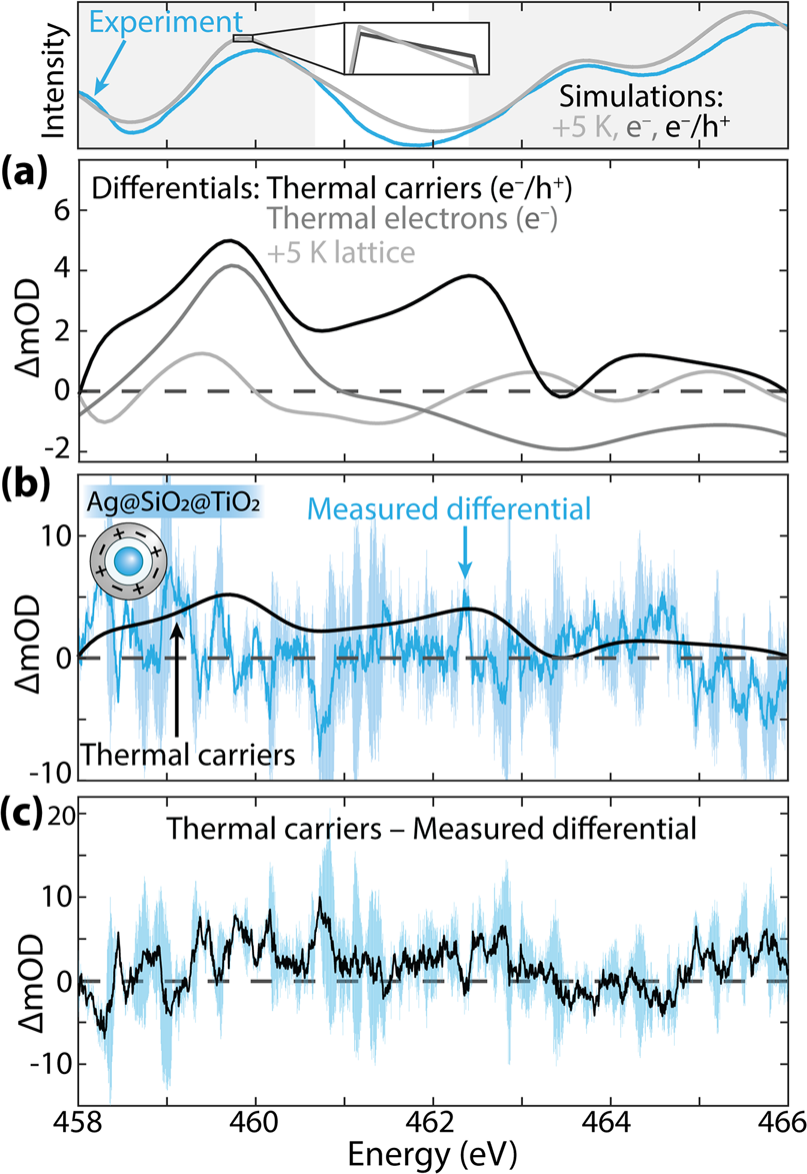
**Thermalized electron plus hole effects on the Ti L**_**2,3**_**edge in Ag@SiO**_**2**_**@TiO**_**2**_**.** (Top) Raw spectra for reference. The spectral range analyzed by the MSE is shaded. (a) Simulated differential spectra for a +5 K lattice expansion (light gray), thermalized electrons (gray), and thermalized carriers (black). The thermalized carriers are simulated at each respective band edge. (b) Thermalized carriers compared to the Ag@SiO_2_@TiO_2_ measured differential spectrum. The blue shaded region depicts the experiment’s standard deviation. (c) The measured differential subtracted from the optimized simulation differential shown in (b) with the standard deviation.

Lastly, the Ag@SiO_2_@TiO_2_ nanoparticle system is examined and is expected to have thermalized electron and hole pairs (Figure 7). This is the most challenging example to model as electrons, holes, and photothermal effects are present within the noisy experimental spectrum, and the hole more weakly perturbs the spectrum than the electron since the probed Ti 2p states predominantly compose the conduction band (Figure 3a). The holes only change the screening and angular momentum components of the core–valence excitons in the X-ray spectrum without adding or blocking new transitions, usually the larger signal. The theoretical differential absorption in Figure 7a as compared to thermal and hot electron changes does demonstrate that adding holes to the calculation should have a measurable effect but experimentally requires a better SNR. Namely, introducing holes to a photoexcited electrons-only model produces an increase in the differential absorption largely at 462 eV and across the spectrum.

The Ag@SiO_2_@TiO_2_ nanoparticles’ measured differential absorption is compared to the simulated differential for thermalized carriers in Figure 7b. Calculating the differential absorption spectra and corresponding MSE for thermalized electrons, thermalized electron–hole pairs, and photothermal effects reveals that there is no statistical difference in the MSE for the three calculations, despite the changes in the spectra between these three photoexcited effects (Figure S2c). The initial spectral features are well-captured in Figure 7b, but this alone is not enough to signify a statistical difference from those of the other models. The photocharging indicates that the plasmonic resonance energy transfer effect is present as in previous reports, but a less noisy spectrum or a higher excitation density, which would exaggerate the pre-edge and hole effects, would be needed to differentiate all three models. We also plot the difference between the measured and calculated differentials in Figure 7c. The amplitude of the residual further reflects that the excited-state calculation is unable to model the experiment. However, Figure 7a indicates that the separation of electrons, holes, or electrons plus holes from photothermal effects should be possible in a quasi-equilibrium photomodulated experiment.

Our theoretical analysis, verified by a comparison to ultrafast experiments (Figure S1), provides valuable guidance for comparing each photoexcited effect’s spectrum to experimental X-ray data. Based on this analysis, future photomodulated spectroscopy measurements should aim for a photomodulated or differential SNR > 2 or an average differential signal of ∼5 mOD. The SNR in this work is 0.5, determined by dividing the average differential signal (1.4 mOD in Figure 6c) by the noise root-mean-squared (2.8 mOD in Figure 6d). Ultrafast studies have larger SNRs due to a higher impulse response, carrier density, and spectral chopping rate frequency, as apparent in Figure S1. Additional synchrotron measurements with more extensive experimental controls would be necessary to accurately quantify the steady-state carrier equilibrium. These measurements could consider the following: a total fluorescence yield detection scheme to avoid sample charging artifacts, power-dependent photoexcitation measurements (to modulate the carrier density amplitude and temperature effects), and rapid photomodulation or extensive light-on/light-off measurements to confirm signal reproducibility. Ideal specimens would be nanometer-scale crystalline semiconductors with long carrier lifetimes or polaronic traps to increase the likelihood that atoms contributing X-ray signal are properly photoexcited (not bulk atoms in the ground state).

## Conclusion

We used a set of plasmonic core–shell nanoparticles to test if a BSE-based analysis can differentiate heating, hot electron, and thermalized carrier effects in quasi-equilibrium photomodulated X-ray absorption experiments. The lattice temperature and hot carrier energy were successfully separated and analyzed within a noisy experimental spectrum. Separating a thermalized electron and hole carrier distribution was not as successful, although this outcome is mainly due to a lower experimental signal and a spectral cancellation unique to the Ti L_2,3_ edge. The BSE method proposed appears accurate enough to allow nontime-resolved X-ray beamlines to determine electron and hole effects, greatly expanding the realm of photoexcited studies. This is a particularly important advance for systems in which defects, hot carrier effects, and junctions that control transport and surface catalysis through steady-state distributions are difficult to study with ultrafast spectroscopy. However, measuring quasi-equilibrium distributions requires careful balance of the excitation rate, recombination rate, and photomodulation time. The results of our paper therefore give technical guidelines for measuring simultaneous electron, hole, and thermal quasi-equilibrium populations. The spectral signatures for each excited-state effect, and their intensity with excitation density, are particularly useful for future steady-state and ultrafast measurements of anatase TiO_2_.

## Methods

### Core–Shell Nanoparticle Synthesis and Characterization

Core–shell metal@(SiO_2_)@TiO_2_ nanoparticles were synthesized and characterized previously, and this work is only a theoretical analysis of X-ray spectra for these same particles.^28^ All experimental X-ray and UV–Vis spectra were collected at the time of these referenced initial publications. Aqueous-phase UV–Vis absorption (Shimadzu 2550) measured the localized surface plasmon resonance and TiO_2_ absorption onset for each particle (Figure S3).

### Photodiode Heterojunction Modeling

A drift-diffusion model is used to simulate the metal–semiconductor junction for each core–shell nanoparticle design through the Automat FOR Simulation of HETerostructures (AFORS-HET) software v.2.5. This numerical simulation software uses a 1D drift-diffusion model based on self-consistent solutions to the Poisson equation to model the band bending, carrier tunneling, and junction properties.^55^ See Supporting Information for input parameters and the built-in field calculation.

### X-ray Absorption Spectroscopy

The National Synchrotron Radiation Research Center (NSRRC) in Hsinchu, Taiwan, collected all Ti L edge X-ray spectra at the BL20A1 beamline in total electron yield mode (reflection geometry), depicted in Figure S4. Each specimen was mounted on conductive Cu tape without surface treatment. All secondary electrons were collected to generate the detected signal. The total Xe lamp power density was ∼200 mW/cm^2^ at the sample and ∼10 mW/cm^2^ across each plasmon resonance energy range. The photon flux was measured 1 m away from the lamp with an initial power of 500 W. The lamp was spectrally filtered to irradiate the sample with >400 nm light or below the TiO_2_ bandgap, and non-AR coated optics were used as the entrance windows. The X-ray spectra were collected with a (lamp off), (lamp on), (lamp off), and (lamp on) sequence for 48 s acquisition per spectrum. The X-ray analysis software at the beamline was used to subtract the background (X-ray scattering and electron emission) by using a straight baseline fit below the absorption rising edge.

### Ab Initio X-ray Theory

The X-ray absorption simulation package, Obtaining Core Excitations from the *Ab initio* electronic structure and the NIST BSE solver (OCEAN), was used to model the plasmonic and excited-state properties in TiO_2_.^56,57^ The package’s workflow has been described previously.^37,38^ A variable-cell relaxation of the TiO_2_ anatase crystal structure was initially performed to define suitable cell parameters. As part of the workflow, Quantum ESPRESSO^58,59^ performed density functional theory (DFT) to calculate the ground-state electronic structure using a plane-wave basis set and a 350 Ry cutoff energy. The DFT used Trouiller-Martins norm-conserving pseudopotentials calculated using a Perdew–Wang local density approximation (LDA). A 16 × 16 × 12 *k*-point mesh was used with 248 total bands. The macroscopic dielectric constant was set to 5.62 for TiO_2_ anatase.^60^ The Haydock solver is used to calculate all X-ray spectra. The spin–orbit coupling for all BSE calculations was fixed at 4.5 eV. See the Supporting Information for input parameters of the cutoff energy convergence, BSE screening, variable-cell relaxation, and band structure calculations.

The standard OCEAN code can interpret static lattice heating by rerunning the DFT and BSE calculations for lattice parameters that simulate an isotropic thermal lattice expansion. However, our previous reports discuss the modified BSE code that simulates excited-state electrons and holes.^26,37,38^ The standard code is modified to output the band structure as a usable *k*-point mesh array with defined energy values for each *k*-point. This array is then evaluated and modified to include an excited-state carrier population through a state filling. Specifically, photoexcited electrons are simulated by blocking available transitions in the conduction band, while holes are simulated by opening or making states available in the valence band. It is worth noting that the state filling fully occupies each state, whereas a partial occupation would more accurately depict a carrier density. We use an iterative approach to state filling in MATLAB, which fills all conduction states up to a specified energy or opens valence states for holes. However, this method is complicated for band structures with degenerate valleys across *k*-space because the *k*-point mesh in OCEAN is unsorted. In other words, degenerate valleys would be unavoidably filled with excited-state electrons and the simulated excitation would not be momentum-specific.

After the X-ray absorption spectra are calculated, they are broadened to account for the experimental lifetime broadening of the TiO_2_ Ti L_2,3_ edge.^36^ The theoretical differential absorption is then calculated as the log of the excited-state spectrum divided by the ground-state spectrum. The mean squared error (MSE) between the calculation and experiment is calculated using MATLAB’s “goodnessOfFit” function. See the Supporting Information for the lattice expansion parameters, state-filling simulations/scripts, spectral broadening procedure, and MSE calculations.

## Supporting Information Available

Comparing this work’s calculated spectra to previously measured ultrafast anatase TiO_2_ X-ray absorption spectra, a quantitative analysis of the ab initio calculations’ mean squared error, UV–visible absorption spectra of the core–shell nanoparticles, heterojunction diode model input parameters, energy-dependent broadening scheme, experimental X-ray spectra and the X-ray instrument geometry, interpretation of carrier excitation and relaxation rates, ground-state calculations/convergence parameters, excited-state theory input parameters, and example OCEAN input files (PDF)
