# Supplementary material for: Determining Quasi-Equilibrium Electron and Hole Distributions of Plasmonic Photocatalysts Using Photomodulated X-ray Absorption Spectroscopy
Source: ACS Nano. 2024 Mar 18;18(13):9344–53. doi: 10.1021/acsnano.3c08181 (PMC10993415; doi:10.1021/acsnano.3c08181)
Supplement: Supplementary file 1 — nn3c08181_si_001.pdf [file nn3c08181_si_001.pdf]

Supporting Information:

Determining Quasi-Equilibrium Electron and Hole Distributions of Plasmonic Photocatalysts Using Photomodulated X-ray Absorption Spectroscopy

Levi Daniel Palmer[1], Wonseok Lee[1], Chung-Li Dong[2], Ru-Shi Liu[3], Nianqiang Wu[4], Scott Kevin Cushing[1,*]

[1]Division of Chemistry and Chemical Engineering, California Institute of Technology, Pasadena, CA 91125, USA.
[2]Department of Physics, Tamkang University, New Taipei City 251301, Taiwan
[3]Department of Chemistry, National Taiwan University and Advanced Research Center for Green Materials Science and Technology, Taipei 10617, Taiwan
[4]Department of Chemical Engineering, University of Massachusetts Amherst, Amherst, MA 01003–9303, United States
*Corresponding author. Email: scushing@caltech.edu

Table of Contents

1. Interpreting Previous Ultrafast X-ray Spectra of Anatase TiO_2. .... *3*

2. Quantifying the Fit between Experiment and Theory using Mean Squared Error (MSE). *4*

3. Core-Shell Nanoparticle UV–Visible Spectra. *5*

4. Photodiode Heterojunction Input Parameters. *5*

5. Energy-Dependent Broadening of Simulated Spectra. *6*

6. Raw, Experimental X-ray Spectra and Charging Effects. *7*

7. Comparing Carrier Excitation and Relaxation Rates in Amorphous TiO_2. .... *8*

8. Ground-State Calculations. *8*

8A. Ground-state DFT (Quantum ESPRESSO): Cutoff energy convergence, variable-cell relaxation, and band structure calculations. 9

8B. Ground-state X-ray theory (OCEAN): BSE, screening, and scissor shift. 10

9. Excited-State X-ray Theory and Statistical Error Calculations: lattice expansion parameters (heating), state-filling simulations, and mean-squared error (MSE) calculations. *11*

9A. Heating (Thermal Lattice Expansion) 11

9B. Hot Electrons Simulated in TiO_2 Anatase 12

9C. Electrons and Holes Simulated in TiO_2 Anatase 13

9D. Mean Squared Error (MSE) Calculations 14

9E. Hot Electron X-ray Differential Spectra with Temperature Included (Used for the MSE Analysis) 15

10. References *16*

Appendix A. Example Quantum ESPRESSO Input Files: *18*

Appendix B. Example OCEAN 2.5.2 Input File: *24*

1. Interpreting Previous Ultrafast X-ray Spectra of Anatase TiO_2.

The excited-state Bethe-Salpeter equation (BSE) approach is tested on previous ultrafast X-ray measurements of anatase TiO_2 collected in ref. 1 (**Figure S1**).[1] This test notably benchmarks the theory's accuracy in modeling carrier distributions and potential discrepancies introduced using the TiO_2 anatase phase approximation. **Figure S1a** compares the Ti $L_{2,3}$ transient spectrum 15 ps before and 1 ps after photoexcitation to the ground-state simulation performed in this work. Femtosecond carrier dynamics in TiO_2 are simpler to model than plasmonic quasi-equilibrium hot carrier distributions because the transient carrier populations relax as a function of time after direct photoexcitation. Direct photoexcitation also avoids complex photothermal and carrier trapping effects occurring in the steady state. **Figure S1b** compares the simulation and experiment for ultrafast X-ray spectra of anatase TiO_2. Fully thermalized electrons and holes are simulated with equal contribution. The spectral features are notably similar but the effects at the L_3 t_{2g} and e_g peaks are under- and overapproximated, respectively. This result indicates a relatively strong agreement between the experiment and theory in the advent of well-defined ultrafast carrier dynamics.

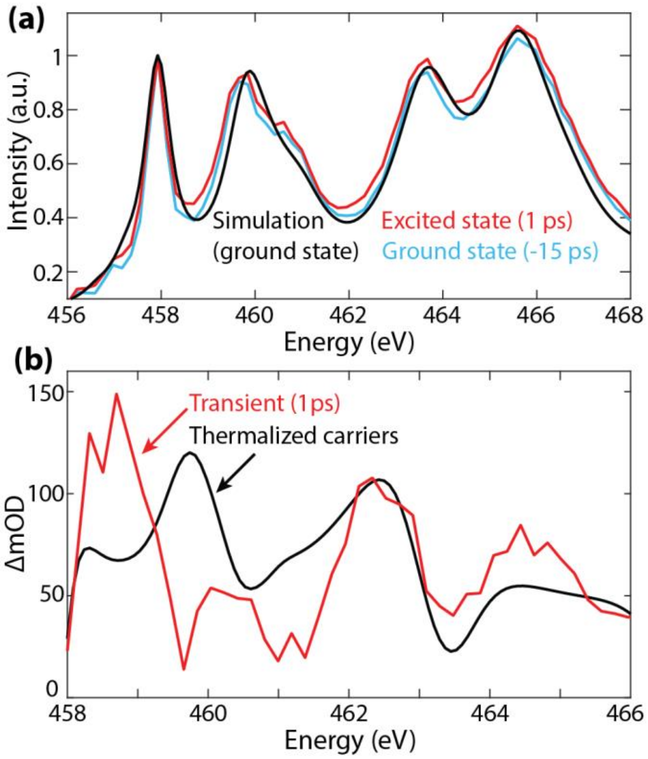

Figure S1. Simulating carriers in ultrafast Ti $L_{2,3}$ edge X-ray spectra of anatase TiO_2. (a) Ground- and excited-state (-15 ps, blue, and 1 ps, red, before/after photoexcitation) experimental spectra of anatase TiO_2 compared to the simulation in this work (black). (b) The 1 ps transient differential of anatase TiO_2 modeled with thermalized electrons and holes using the *ab initio* approach. All experimental spectra were measured in ref. [1].

2. Quantifying the Fit between Experiment and Theory using Mean Squared Error (MSE)

To quantify the agreement between the simulated and experimental spectra, the mean squared error (MSE) is calculated for each simulated spectrum. The MSE, $\frac{\sum_x (y_{simulated} - y_{measured})^2}{N}$, is given by the difference in the spectral intensity for the simulated ($y_{simulated}$) and measured ($y_{measured}$) spectra at each point in energy normalized to the total number of analyzed points in the spectrum (N). The MSE represents the average squared difference between the experiment and theory across the spectral range.

For Au@SiO_2@TiO_2, the differential X-ray spectrum for each simulated lattice temperature (ΔT of 0, 2.5, 5.0, 10, 15, and 20 K) was calculated, as shown in **Figure S2a**. The MSE for each temperature was fit using a quadratic regression model (R^2 = 0.99) to determine the lattice temperature of the Au@SiO_2@TiO_2 nanoparticles. Because the quadratic regression fits the quadratic relationship of the MSE (mOD^2) as a function of temperature (K), the vertex reflects the best match between the experiment and theory, giving an estimate of the photothermal temperature. The fit minimum at 2.5 K has a MSE of ~5 mOD^2, or the average difference between the simulated and measured spectrum is ~2 mOD across the spectral range, rounding to the first significant digit.

For Au@TiO_2, the MSE prediction and quadratic regression model (R^2 = 0.95) were again used to determine the most likely combination of heating and hot electron occupation. The ~5 mOD^2 MSE minimum in the quadratic regression suggests that hot electrons exist up to 0.3 eV above the CBM when including a 14 K lattice expansion, where both temperature and electron energy are optimized by minimizing the total MSE (**Figure S2b**). This MSE again equates to an average difference of ~2 mOD between the simulated and measured spectra.

For Ag@SiO_2@TiO_2, the MSEs for a +5 K lattice expansion, thermalized electrons, and thermalized carriers are displayed. There was no determined significant difference between the three simulations although thermalized electron-hole pair reportedly exist in the TiO_2 layer.

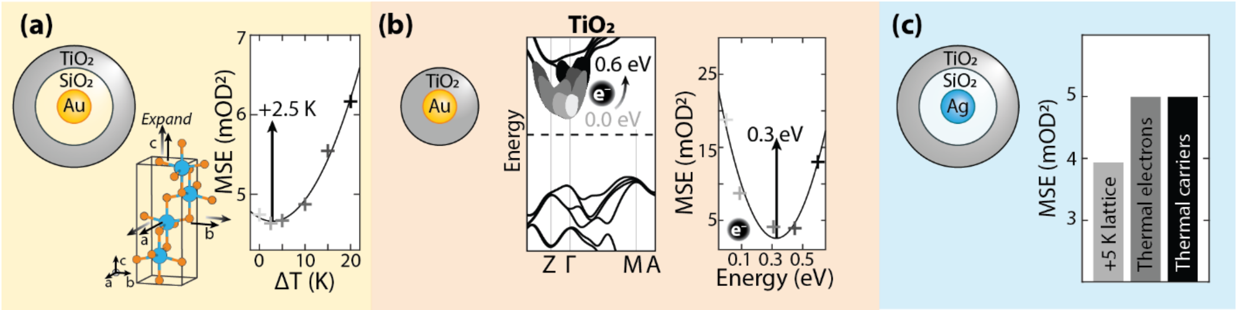

Figure S2. Mean squared error (MSE) analysis for the metal@(SiO_2)@TiO_2 nanoparticle X-ray simulations. The MSE between the simulated and measured spectra in Figures (a) 5, (b) 6, and (c) 7 within the main text. The MSEs in (a) and (b) are fit to a quadratic regression model with the fit's vertex at 2.5 K and 0.3 eV, respectively. The MSE analysis in (b) contains both the hot electron simulation and a 14 K lattice expansion as shown by the spectra in **Figure S11**.

3. Core-Shell Nanoparticle UV–Visible Spectra.

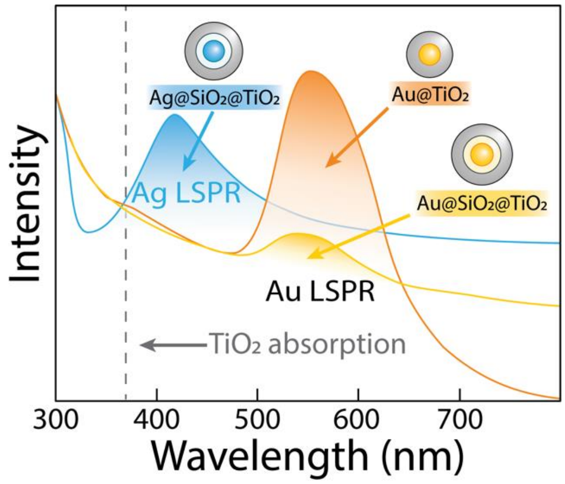

Figure S3. UV–visible absorption spectra for the metal@(SiO_2)@TiO_2 nanoparticles. The intensity is scaled to the TiO_2 absorption onset to depict the relative position between each localized surface plasmon resonance (LSPR) and the TiO_2 absorption.

4. Photodiode Heterojunction Input Parameters.

Table S1. Input Heterojunction Materials Properties

Parameter	Units	Ag	Au	SiO_2	TiO_2
Thickness	[nm]	15	15	10	20
Dielectric constant	[–]	1200	1200	11.9	11.9
Electron affinity	[eV]	–	–	1.0-2.0*	4.3
Fermi Level	[eV]	-5.3	-5.2	–	-4.68 (calc.)
Band gap	[eV]	0.001	0.001	9.1	3.2
Cond./val. band density	[cm^{-3}]	1E22/1E22	1E22/1E22	2.9E19/2.7E19	1E21/1E21
Electron/hole mobility	[cm^2/Vs]	1107/424.6	1107/424.6	0.01/0.001	0.1/0.001
Acceptor/donor concentration	[cm^{-3}]	–	–	0/100*	0/5E14
Electron/hole thermal velocity	[cm/s]	1E7/1E7	1E7/1E7	1E7/1E7	1E7/1E7
Layer density	[g*cm^{-3}]	10.5	19.3	2.7	3.84

*The electron affinity and dopant concentration of SiO_2 should be 0.8 eV and 0 cm^{-3}, respectively. However, different values were used to avoid calculation instabilities from the conduction band approaching the vacuum level.

Table S2. Output Heterojunction Results				
**Result**	**Units**	**Au@SiO_2@TiO_2**	**Au@TiO_2**	**Ag@SiO_2@TiO_2**
Schottky barrier	[eV]	0.73 (SiO_2@TiO_2 interface)	0.94	0.79 (SiO_2@TiO_2 interface)
Junction Fermi level	[eV]	-5.3	-5.2	-5.3
Built-in field (in dielectric)	[V/cm]	1.7E+05	2.6E+05	2.1E+05

We calculate the average built-in electric field (F) by calculating the difference in the metal and TiO_2 Fermi levels before forming a junction and dividing this difference by the total dielectric and semiconductor thickness (t) for TiO_2 and SiO_2 following equation S1:

$$F = (E_{F,TiO_2} - E_{F,metal})/t \tag{S1}$$

5. Energy-Dependent Broadening of Simulated Spectra.

The OCEAN code uniformly broadens all simulated X-ray spectra using a set energy broadening input. The calculations in this work include 0.1 eV broadening for each output X-ray absorption spectrum. The calculated spectrum from OCEAN is then manually broadened in MATLAB to account for the inherent lifetime broadening of the core-level X-ray transition. This lifetime, or energy-dependent, broadening is a result of the energy-dependent loss function and the inelastic mean free path of the core electron and core hole in TiO_2. The spectra are manually broadened by convoluting them with Lorentzian functions with 1.05 (L_3 t_{2g}), 1.55 (L_3 e_g), 1.85 (L_2 t_{2g}), and 2.25 (L_2 e_g) eV bandwidths. The average broadening of 2.05 and 1.3 eV for the L_2 and L_3 edges has a ratio of 3:1.9, comparable to the reported 3:2 lifetime broadening ratio for TiO_2 anatase.

To accurately model the intensity of the differential spectral features, the intensity of the calculated spectra was also manually modified in MATLAB. The intensity of the convoluted Lorentzian function during the spectral broadening was manually adjusted for each major peak in the experiment to match the ground-state intensity. These peaks include the t_{2g} and e_g peaks for both the L_3 and L_2 edges. The relative intensity multiplier used for each peak was 1 (L_3 t_{2g}), 1.65 (L_3 e_g), 1.35 (L_2 t_{2g}), and 2.76 (L_2 e_g) a.u.

The post-edge region was also manually broadened by 5.0 eV and amplified by 3 a.u. However, the differential spectrum in this region was not interpreted due to the sample charging in the

experimental measurements. This large (and somewhat arbitrary) manual modification of the post-edge region is a testament to the fact that OCEAN models core-level features on/before the edge better than post-edge features and extended X-ray absorption fine-structure.

6. Raw, Experimental X-ray Spectra and Charging Effects.

All experimental data was collected at the BL20A1 beamline (National Synchrotron Radiation Research Center in Hsinchu, Taiwan) in total electron yield mode (reflection geometry) depicted in **Figure S4**.

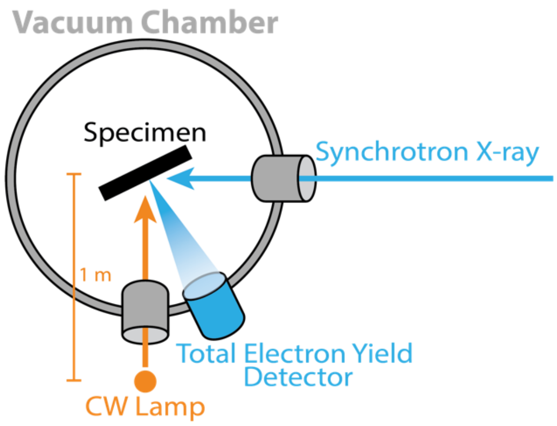

Figure S4. Photomodulated X-ray absorption spectroscopy in total electron yield detection geometry. A continuous-wave (CW) lamp photoexcites the specimen from 1 meter away. The incident X-rays probe the dynamics with the lamp on/off, and a total electron yield detector collects electron scattering to emulate X-ray absorption spectroscopy.

The raw, experimental data show spectral intensity fluctuations due to charging for both Au@TiO_2 and Ag@SiO_2@TiO_2 (**Figure 4a**). The influence of charging is apparent by the greater intensity of the light on spectrum (orange or blue) compared to the light off spectrum (black). The Au@SiO_2@TiO_2 nanoparticles do not reflect charging signatures, which validates the control by suggesting the SiO_2 layer effectively blocks hot electron transfer from Au to TiO_2. Sample charging is measurable in the total electron yield geometry because the surface charging causes the detected electrons to be acquired more or less efficiently, depending on the charge type, due to Coulombic repulsion.

Normalization: The spectra in **Figure 4a** were normalized to the X-ray edge onset maximum near 458 eV to correct for the spectral charging artifacts (**Figure 4b**). This is indicated by a grey dashed line to guide the eye. Using the normalized spectra, the differential absorption was calculated by $\Delta \mathrm{mOD} = \log_{10}\left(\frac{\text{light on}}{\text{light off}}\right) * 10^3$ for each of the two data sets (**Figure 4c**) and averaged (**Figure S5**). The spectra were only interpreted between 458 and 466 eV to avoid regions that may have

been affected by the charging normalization. These spectra are overlaid to have a better direct comparison of the differential features and intensities (**Figure S5**).

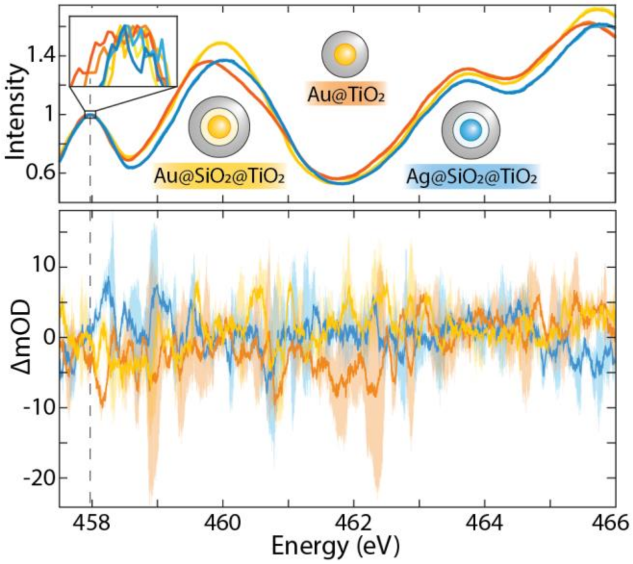

Figure S5. Photomodulated X-ray spectra of the Ti $L_{2,3}$ edge. (Top) Experimental ground-state and photomodulated Ti $L_{2,3}$ edge spectra and (Bottom) differential spectra for each core-shell nanoparticle system: Au@SiO_2@TiO_2 (yellow), Au@TiO_2 (orange), and Ag@SiO_2@TiO_2 (blue). The ground-state spectra are depicted in a lighter shade of each color, but are only distinguishable in the inset. The differential solid lines depict the average raw differential spectra, and the shading depicts the standard deviation of each data point across two averaged spectra. The grey dashed line denotes the point used for charging (amplitude) normalization.

7. Comparing Carrier Excitation and Relaxation Rates in Amorphous TiO_2.

With the experiment's 10 mW/cm^2 power density (2.7*10^{16} photons/(s*cm^2) at 560 nm) and the reported ~45% injection efficiency of hot electrons from Au into TiO_2, roughly 1.2*10^{16} hot electrons/(s*cm^2) inject into TiO_2.[2] This corresponds to ~6*10^5 hot electrons/s injected into each particle's TiO_2 layer, assuming uniform particle packing. Ultrafast measurements indicate that hot electrons in crystalline TiO_2 films fully thermalize within 20 – 50 fs,[3] but other steady-state spectroscopic measurements have reported hot electron trapping in amorphous TiO_2 surface states that prevents carrier and phonon scattering and extends the carrier cooling time.[4–6]

8. Ground-State Calculations.

8A. Ground-state DFT (Quantum ESPRESSO): Cutoff energy convergence, variable-cell relaxation, and band structure calculations.

Quantum ESPRESSO (QE), a DFT package, was used to calculate the ground-state electronic structure inputs for the OCEAN X-ray calculations. A variable-cell crystal structure relaxation was used to define the simulated atomic coordinates of TiO_2 anatase, and a convergence calculation in QE was used to define the cutoff energy. QE was also separately used to calculate the projected density of states (PDOS) and band structure of TiO_2.

Variable-Cell Relaxation. A variable-cell relaxation (vc-relax) calculation was used to determine the anatase atomic coordinates by optimizing the unit cell dimensions. The vc-relax calculation was completed with the cell_dofree = 'ibrav' input to maintain consistency in the lattice structure while relaxing (optimizing) the unit cell axis and angles. See **Appendix A** for input parameters.

Convergence Calculations. The cutoff energy used for the X-ray simulations was first checked for total energy convergence using QE self-consistent field (SCF) calculations. The SCF calculation (**Appendix A**) calculates the total energy of the unit cell lattice, which is used to determine the calculation's convergence. The convergence is a representation of the decrease in the total energy and a higher calculation accuracy following the increase of the wavefunction's (pseudopotential's) energy. The purpose is to preserve calculation accuracy while reducing computational expense. As shown in **Figure S6**, the 350 Ry cutoff energy used for all OCEAN calculations was sufficiently converged.

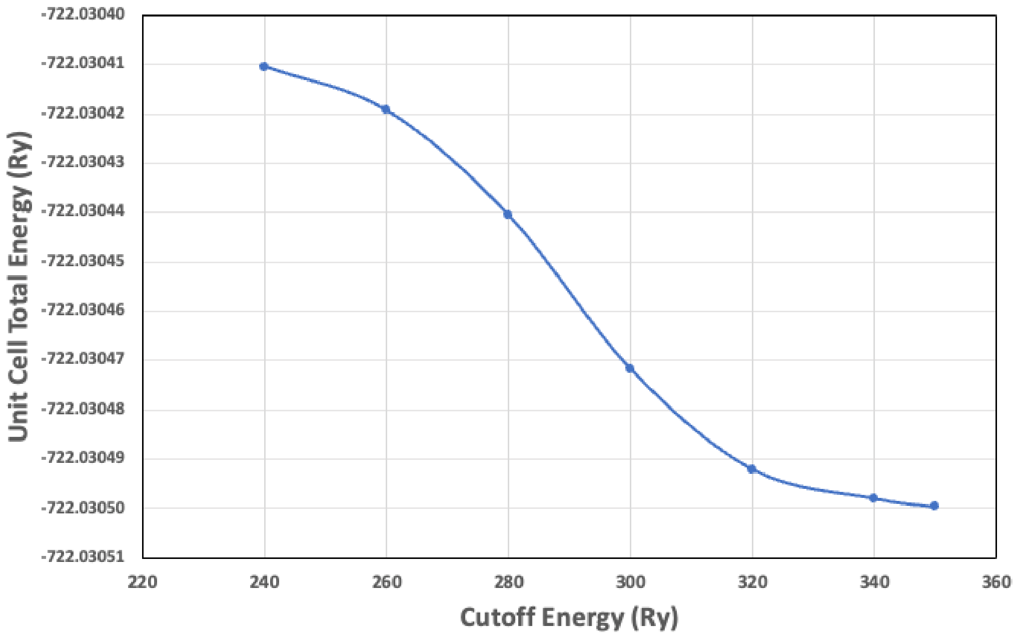

Figure S6. Cutoff energy convergence. A TiO_2 (anatase) self-consistent field calculation was used to calculate the total unit cell lattice energy at various QE cutoff energy input values.

The k-point mesh convergence was similarly confirmed using the 350 Ry cutoff energy. However, the k-points largely determine the accuracy of the state-filling calculation, so a large k-point mesh are desired regardless of the convergence threshold. A 16x16x12 k-point mesh was used for all OCEAN X-ray calculation (**Appendix B**).

Projected Density of States (PDOS) and Band Structure. The band structure simulation included the following calculation stages: SCF, non-self-consistent field (NSCF), Bands, and converting/plotting the data. The SCF calculation calculates the wavefunctions for the unit cell used for the density of states calculation, which is extrapolated in k-space with the NSCF calculation using a higher k-point mesh. The input parameters for each step can be found in **Appendix A**. Plotted in **Figure S7B**, the band structure k-path is defined in the Bands calculation, following previous literature.[7] The calculated Fermi level is located at the valence band edge when calculated with DFT.

The PDOS simulation included the following calculation stages: SCF, NSCF, and plotting the projected states for each atomic orbital. The PDOS is plotted in **Figure S7C** for the relevant valence states, Ti 3d (blue) and O 2p (orange). Background shading in **Figure S7C,D** depicts the crystal field-split t_{2g} (blue shading) and e_g (grey shading) states.

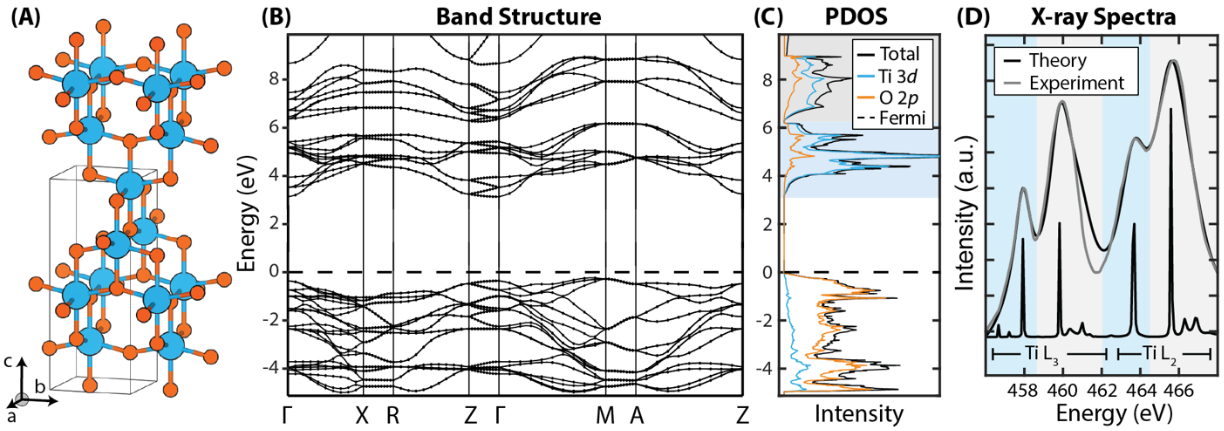

Figure S7. *Ab initio* ground-state calculations of TiO_2 anatase. (A) The variable-cell relaxed crystalline TiO_2 anatase structure unit cell with calculations of the (B) band structure, (C) projected density of states (PDOS), and (D) X-ray spectra. A 1 eV scissor shift is applied to extend the bandgap to the experimental value. The band structure and PDOS were calculated using Quantum ESPRESSO, and the calculated X-ray spectra were calculated with OCEAN and are also shown in the main text.

8B. Ground-state X-ray theory (OCEAN): BSE, screening, and scissor shift.

To simulate the TiO_2 (anatase) $L_{2,3}$ edge, the 'Obtaining Core Excitations from *Ab initio* electronic structure and the NIST BSE solver (OCEAN)' code was implemented. OCEAN is a DFT and

GW/BSE approach to simulating core-level electron excitations. The DFT framework is the Quantum ESPRESSO package,[8,9] specified in the OCEAN input file (see **Appendix B**). The DFT stage is first calculated in OCEAN to determine the ground-state electronic structure.

Notably, the OCEAN code uses the BSE to simulate the Coulombic effects of the core-to-valence transition exciton. OCEAN implements a screening stage to simulate the screening of the core-hole by the valence state electrons. The combined BSE and screening stages alongside the angular momentum matrix elements of the X-ray transition Hamiltonian calculate the core-level transition (or X-ray absorption) spectrum.

An additional input, core_offset = .true., was included in the OCEAN calculations here to calculate the core-level shifts. The core-level shifts are important because they produce the Kohn-Sham potentials at each atomic site, which improves the calculation's accuracy by accounting for the unique core-level shifts at each atom and screening radius even if the atoms are equivalent sites. A 1 eV scissor shift was also used to adjust the simulated band gap to the 3.2 eV experimental value, but this additional step did not appear to significantly affect the output X-ray spectrum.

9. Excited-State X-ray Theory and Statistical Error Calculations: lattice expansion parameters (heating), state-filling simulations, and mean-squared error (MSE) calculations.

An adiabatic approximation was taken to simulate the photoexcited and quasi-equilibrium dynamics. In other terms, the excited-state dynamics are longer lived than the initial electron field excitation from the photon field.

9A. Heating (Thermal Lattice Expansion)

Photoexcited thermal effects are accounted for using the thermal expansion coefficient of TiO_2 anatase.[10] The lattice expansion is anisotropic with the two expansion coefficients being 4.469E-06 K^{-1} (a and b directions) and 8.4283E-06 K^{-1} (c direction). Equation S2 describes the calculation of an expanded lattice parameter (d) at an elevated temperature (T) using the lattice expansion coefficient (α) with an assumed ground-state/room temperature at 300 K. The initial lattice constants at 300 K (d_0) were 7.052 Bohr (a and b directions) and 17.81 Bohr (c direction).

$$d = \left(\alpha * d_0 * (T - 300\ K)\right) + d_0 \qquad \text{(S2)}$$

The lattice expansion was calculated and simulated for 302.5 K, 305 K, 310 K, 315 K, and 320 K. The percent expansion for each temperature is shown in **Table S3**. The spectra calculated with these OCEAN input lattice constants are found in the main text **Figure 5a**.

Table S3: TiO_2 Anatase Lattice Expansion Calculation Results					
**Temperature (K)**	**a (Bohr)**	**b (Bohr)**	**c (Bohr)**	**a % change**	**c % change**
300	7.05288	7.05289	17.81326	–	–
302.5	7.05296	7.05297	17.81363	0.0011	0.0021
305	7.05304	7.05305	17.81401	0.0022	0.0042
310	7.05320	7.05321	17.81476	0.0044	0.0084
315	7.05336	7.05337	17.81551	0.0067	0.0126
320	7.05352	7.05352	17.81626	0.0089	0.0168

9B. Hot Electrons Simulated in TiO_2 Anatase

The OCEAN v2.5.2 source code was previously modified to include photoexcited carriers using state-filling simulations.[11] Following these modifications, the OCEAN code automatically outputs an array file after the CNBSE stage that contains unsorted valence states in k-space defined as occupied (contains an electron = 1) or unoccupied (no electrons = 0). These states are the valence band and conduction band, respectively, and the number of states is set using the *k*-point mesh defined in the OCEAN input file (16x16x12 = 3072 states in this work). **Figure S8A,B** depict the array file output from the OCEAN code where the *k*-points are not sorted along a specific *k*-path following high-symmetry *k*-points. Each *k*-point has an associated energy as shown in **Figure S8A**. Then, the *k*-points can be manually sorted into the same high-symmetry path used for the band structure (**Figure S8C**). Sorting the *k*-points into the band structure is an essential step for later visualizing the simulated carrier distributions.

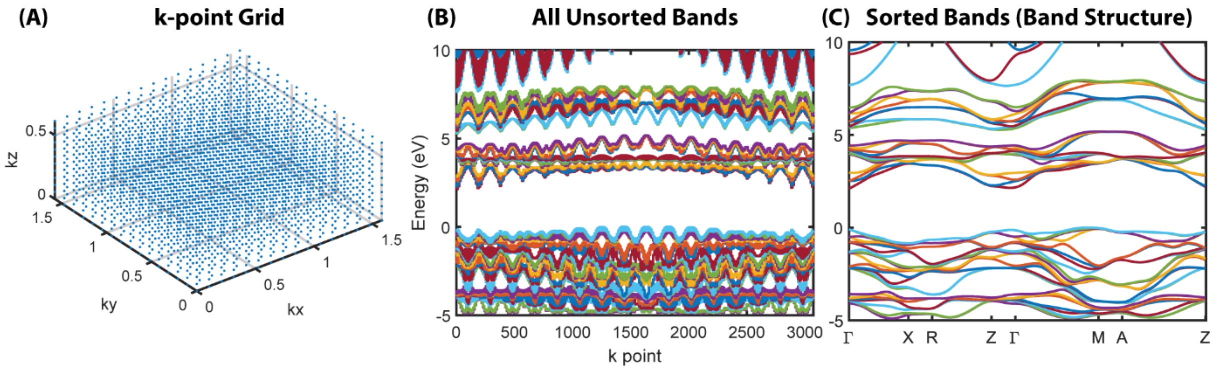

Figure S8. Unsorted and sorted *k*-points for the array file and TiO_2 anatase band structure. (a) The *k*-point grid depicts the number of *k*-points simulated in reciprocal space. (b) The unsorted *k*-points plotted by energy. The 1 eV scissor shift of the band gap is not included, and the Fermi level (0 eV) is defined at the valence band edge. (C) The sorted *k*-points along the *k*-path of the labelled high-symmetry *k*-points.

The state-filling model takes the valence states defined as '0' or '1' and redefines their values to simulate photoexcited electrons and/or holes. For the hot electron state-filling algorithm in this work, the electronic states up to a specified value are all fully filled. Hot electrons up to 0.0 (fully thermalized), 0.1, 0.3, 0.45, and 0.6 eV above the conduction band minimum were simulated in this work, shown in the main text. The input hot electron state-filling simulations used for all calculations are shown in **Figure S9**. The electron distributions were interpolated onto the band structure because OCEAN uses the unsorted *k*-points as an input (**Figure S8B**). Note that this state-filling method would not typically be *k*-point selective for other band structures; however, all conduction band states at or below 0.6 eV for TiO_2 anatase have occupations at or near the gamma point. Therefore, the band structure allows for the simulation of hot electrons at the gamma point alone as compared to other, more complicated band structures that would distribute the hot electrons in *k*-space.

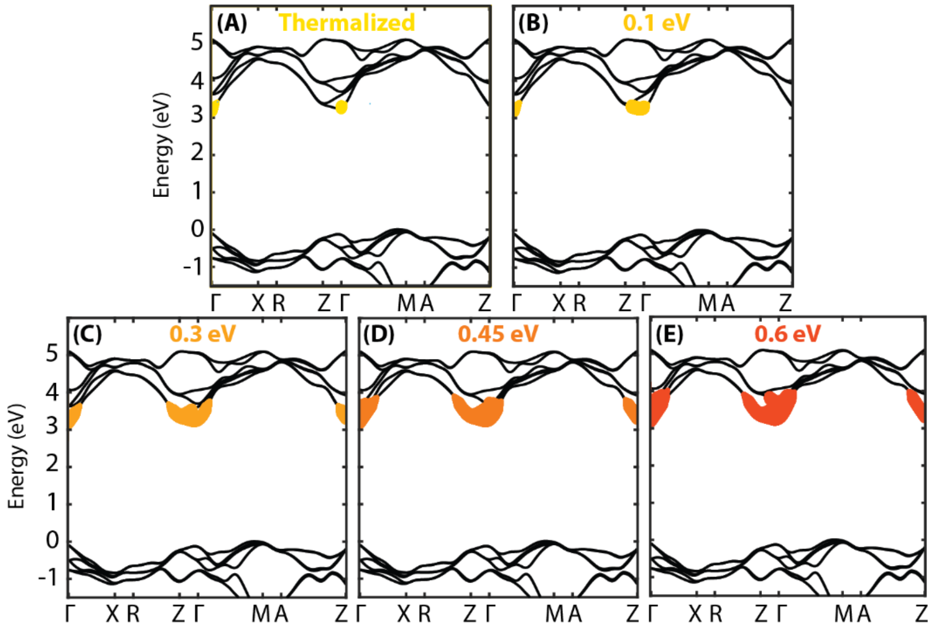

Figure S9. **Hot electron state-filling simulations**. Electronic states simulated with hot electrons (colored in yellow to orange gradient) filling from the conduction band minimum up to (A) 0.0 eV thermalized/one conduction state filled, (B) 0.1 eV, (C) 0.3 eV, (D) 0.45 eV, and (E) 0.6 eV. Each state-filling occupation was separately input into OCEAN. A 1 eV scissor shift is included to accurately depict the simulation input. The bands were interpolated in *k*-space to project the simulated state-filling onto the band structure.

9C. Electrons and Holes Simulated in TiO_2 Anatase

Similar to the procedure for hot electrons alone, fully thermalized electrons and holes were simulated with OCEAN state-filling at the conduction band minimum and valence band maximum. This approach was taken to simulate the effect of plasmon-induced dipole coupling on the photomodulated X-ray spectra. The energies of the thermalized electrons and holes were at the

conduction and valence band edges (only one state filled). **Figure S10** depicts the state-filling simulation with the electrons and holes in each respective band. The M → Γ indirect transition is assumed due to the indirect band gap and lowest transition energy for anatase TiO_2.

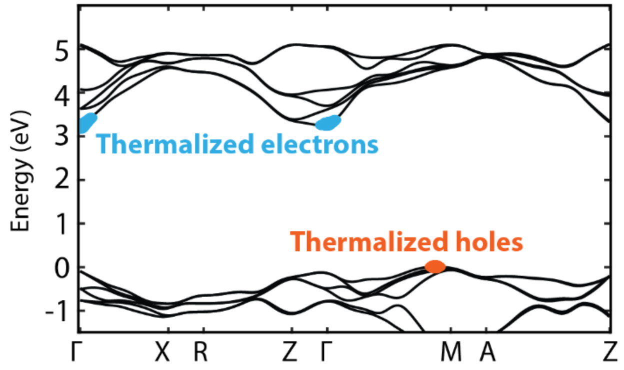

Figure S10. **Fully thermalized electron and hole state-filling simulations.** Thermalized electrons and holes were input into the OCEAN simulations as the state-filled band structure shown. A 1 eV scissor shift is included to accurately depict the simulation input. The bands were interpolated in *k*-space to project the simulated state-filling onto the band structure.

9D. Mean Squared Error (MSE) Calculations

The MATLAB mean squared error (MSE) 'goodnessOfFit' function was used to quantify the difference between the simulated differential spectra from OCEAN and the raw, photomodulated experimental data. Specifically, both the simulated and experimental differential spectra were compared in the 458 – 460.6 eV and 462.4 – 466 eV energy ranges of interest using the 'MSE' fit function. These energy windows were selected because of the accuracy of the TiO_2 anatase approximation for the experimentally measured amorphous TiO_2 (**Figure 3**). For the lattice heating simulations, the intensity of the differential spectrum was unmodified and determined solely by the intensity of the simulated X-ray spectrum output. For the state-filling simulations, the intensity of the spectrum was first chosen to minimize the MSE between the simulated and experimental X-ray differential spectra. This is essential because the OCEAN code cannot accurately predict the exact differential intensity (ΔOD) of carriers as partial state occupations, and a low carrier density exists experimentally. The state-filling simulation's differential intensity was then normalized to the number of simulated carriers to account for intensity fluctuations caused by the increased carrier density. The values selected for normalizing the hot electron state-filling spectral intensity are shown in **Table S4**, and the total intensity of the spectrum per carrier simulated is 4,500,000.

Table S4: TiO_2 Anatase State-Filling Spectrum Normalization		
**Number of Simulated States Filled**	**Spectrum Multiplier**	**Total Intensity per Carrier Simulated**
1 (Thermalized at CBM)	400000	400000*
20 (~0.1 eV above CBM)	200000	4000000
150 (~0.3 eV above CBM)	26666	4000000
300 (~0.45 eV above CBM)	13333	4000000
566 (0.65 eV above CBM)	7067	4000000

*The intensity for one (1) simulated electron was found to be notably highly non-linear as compared to the intensity for 20 – 566 electrons in the conduction band. The total carrier intensity multiplier was arbitrarily set to 400,000 for this fully thermalized electron simulation.

After the MSE was calculated for each simulation, the lattice heating and hot electron state-filling simulations were further examined using a quadratic regression of the MSEs to define the optimal/minimized lattice temperature and hot electron energy. MATLAB's 'fitlm' function and a 'quadratic' regression model were used to determine the R-squared value of the quadratic fit, and the function's minimum was referenced as the temperature or hot electron energy most representative of the experiment's conditions.

9E. Hot Electron X-ray Differential Spectra with Temperature Included (Used for the MSE Analysis)

Following the hot electron X-ray simulations, a 14 K thermal contribution to the spectrum was included. This lattice heating was added for the MSE analysis, which is not reflected in **Figure 6b** in the main text. For clarity and transparency, these simulated differentials are presented in **Figure S11**.

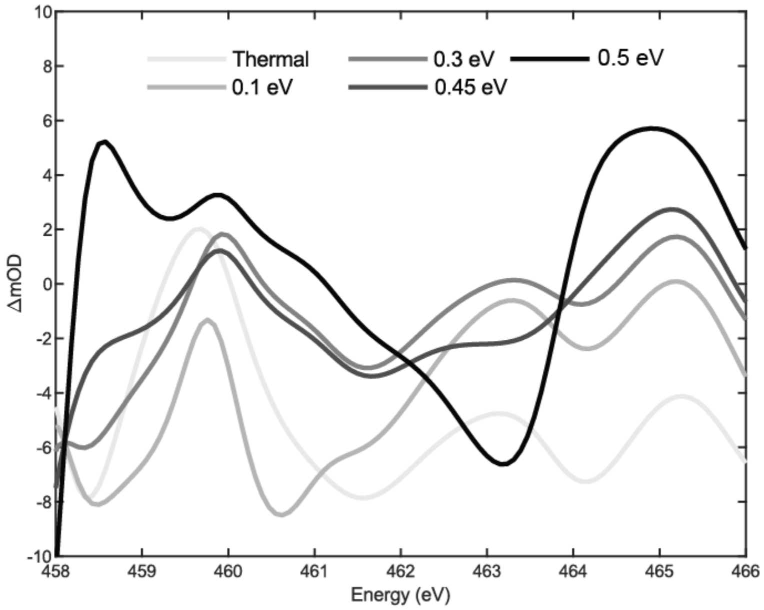

Figure S11. **Simulated hot electron differential spectra with temperature included.** The temperature incorporated in the simulation was fixed at 13.5 K with a 5.8 mOD intensity shift (y-axis) and the carrier normalization as highlighted in **Table S4**.

10. References

(1) Park, S. H.; Katoch, A.; Chae, K. H.; Gautam, S.; Miedema, P.; Cho, S. W.; Kim, M.; Wang, R.-P.; Lazemi, M.; de Groot, F.; Kwon, S. Direct and Real-Time Observation of Hole Transport Dynamics in Anatase TiO_2 Using X-ray Free-Electron Laser. *Nat Commun* **2022**, *13* (1), 2531. https://doi.org/10.1038/s41467-022-30336-1.

(2) Ratchford, D. C.; Dunkelberger, A. D.; Vurgaftman, I.; Owrutsky, J. C.; Pehrsson, P. E. Quantification of Efficient Plasmonic Hot-Electron Injection in Gold Nanoparticle–TiO_2 Films. *Nano Lett.* **2017**, *17* (10), 6047–6055. https://doi.org/10.1021/acs.nanolett.7b02366.

(3) Tan, S.; Argondizzo, A.; Ren, J.; Liu, L.; Zhao, J.; Petek, H. Plasmonic Coupling at a Metal/Semiconductor Interface. *Nature Photon* **2017**, *11* (12), 806–812. https://doi.org/10.1038/s41566-017-0049-4.

(4) Priebe, J. B.; Karnahl, M.; Junge, H.; Beller, M.; Hollmann, D.; Brückner, A. Water Reduction with Visible Light: Synergy between Optical Transitions and Electron Transfer in Au-TiO_2 Catalysts Visualized by In Situ EPR Spectroscopy. *Angewandte Chemie International Edition* **2013**, *52* (43), 11420–11424. https://doi.org/10.1002/anie.201306504.

(5) Amidani, L.; Naldoni, A.; Malvestuto, M.; Marelli, M.; Glatzel, P.; Dal Santo, V.; Boscherini, F. Probing Long-Lived Plasmonic-Generated Charges in TiO_2/Au by High-Resolution X-ray Absorption Spectroscopy. *Angewandte Chemie International Edition* **2015**, *54* (18), 5413–5416. https://doi.org/10.1002/anie.201412030.

(6) Zhang, Y.; He, S.; Guo, W.; Hu, Y.; Huang, J.; Mulcahy, J. R.; Wei, W. D. Surface-Plasmon-Driven Hot Electron Photochemistry. *Chem. Rev.* **2018**, *118* (6), 2927–2954. https://doi.org/10.1021/acs.chemrev.7b00430.

(7) Landmann, M.; Rauls, E.; Schmidt, W. G. The Electronic Structure and Optical Response of Rutile, Anatase and Brookite TiO_2. *J. Phys.: Condens. Matter* **2012**, *24* (19), 195503. https://doi.org/10.1088/0953-8984/24/19/195503.

(8) Giannozzi, P.; Baroni, S.; Bonini, N.; Calandra, M.; Car, R.; Cavazzoni, C.; Ceresoli, D.; Chiarotti, G. L.; Cococcioni, M.; Dabo, I.; Corso, A. D.; Gironcoli, S. de; Fabris, S.; Fratesi, G.; Gebauer, R.; Gerstmann, U.; Gougoussis, C.; Kokalj, A.; Lazzeri, M.; Martin-Samos, L.; Marzari, N.; Mauri, F.; Mazzarello, R.; Paolini, S.; Pasquarello, A.; Paulatto, L.; Sbraccia, C.; Scandolo, S.; Sclauzero, G.; Seitsonen, A. P.; Smogunov, A.; Umari, P.; Wentzcovitch, R. M. QUANTUM ESPRESSO: A Modular and Open-Source Software Project for Quantum Simulations of Materials. *J. Phys.: Condens. Matter* **2009**, *21* (39), 395502. https://doi.org/10.1088/0953-8984/21/39/395502.

(9) Giannozzi, P.; Andreussi, O.; Brumme, T.; Bunau, O.; Buongiorno Nardelli, M.; Calandra, M.; Car, R.; Cavazzoni, C.; Ceresoli, D.; Cococcioni, M.; Colonna, N.; Carnimeo, I.; Dal Corso, A.; de Gironcoli, S.; Delugas, P.; DiStasio, R. A.; Ferretti, A.; Floris, A.; Fratesi, G.; Fugallo, G.; Gebauer, R.; Gerstmann, U.; Giustino, F.; Gorni, T.; Jia, J.; Kawamura, M.; Ko, H.-Y.; Kokalj, A.; Küçükbenli, E.; Lazzeri, M.; Marsili, M.; Marzari, N.; Mauri, F.; Nguyen, N. L.; Nguyen, H.-V.; Otero-de-la-Roza, A.; Paulatto, L.; Poncé, S.; Rocca, D.; Sabatini, R.; Santra, B.; Schlipf, M.; Seitsonen, A. P.; Smogunov, A.; Timrov, I.; Thonhauser, T.; Umari, P.; Vast, N.; Wu, X.; Baroni, S. Advanced Capabilities for Materials Modelling with Quantum ESPRESSO. *J Phys Condens Matter* **2017**, *29* (46), 465901. https://doi.org/10.1088/1361-648X/aa8f79.

(10) Hummer, D. R.; Heaney, P. J.; Post, J. E. Thermal Expansion of Anatase and Rutile between 300 and 575 K Using Synchrotron Powder X-ray Diffraction. *Powder Diffraction* **2007**, *22* (4), 352–357. https://doi.org/10.1154/1.2790965.

(11) Liu, H.; Michelsen, J. M.; Mendes, J. L.; Klein, I. M.; Bauers, S. R.; Evans, J. M.; Zakutayev, A.; Cushing, S. K. Measuring Photoexcited Electron and Hole Dynamics in ZnTe and Modeling Excited State Core-Valence Effects in Transient Extreme Ultraviolet Reflection Spectroscopy. *J. Phys. Chem. Lett.* **2023**, *14* (8), 2106–2111. https://doi.org/10.1021/acs.jpclett.2c03894.

Appendix A. Example Quantum ESPRESSO Input Files:

Self-Consistent Field Input File (example used for band structure calculation):

```
&control
    calculation = 'scf'
    restart_mode='from_scratch'
    prefix = 'anatase'
    outdir = './outdir'
    pseudo_dir = '-'
/
&system
  ibrav = 0
  nat = 12
  ntyp = 2
  noncolin = .false.
  lspinorb = .false.
  ecutwfc = 220
  occupations = 'fixed'
  smearing = 'gaussian'
  degauss = 0.002
  nspin  = 1
  tot_charge  = 0.0
  nosym = .true.
  noinv = .true.
/
&electrons
  conv_thr = 1.1d-8
  mixing_beta = 0.3
  electron_maxstep = 250
  startingwfc = 'atomic+random'
  diagonalization = 'david'
/
ATOMIC_SPECIES
 Ti  47.86700  ti.fhi.UPF
 O   15.99940  08-o.lda.fhi.UPF
CELL_PARAMETERS cubic
   7.052884613    0.000000000  0.0000000000
   0.000000000    7.052893877  0.0000000000
   0.000000000    0.000000000  17.813258477
ATOMIC_POSITIONS crystal
Ti     0.9989304300     0.9990218340     0.9889160540
Ti     0.4989346150     0.4990157270     0.4889157940
Ti     0.9989331860     0.4990225240     0.2389181510
Ti     0.4989289910     0.9990164210     0.7389132230
O     0.9989302620     0.9990225790     0.1969983610
```

```
O     0.4989269390     0.4990168940     0.6969918920
O     0.9989346180     0.4990128120     0.4469955950
O     0.4989312380     0.9990212260     0.9469954940
O     0.4989334320     0.4990235570     0.2808392350
O     0.9989301330     0.9990146590     0.7808332590
O     0.4989345910     0.9990164110     0.5308359080
O     0.9989308730     0.4990219780     0.0308359370

K_POINTS (automatic)
  6 6 6 0 0 0
```

Non-Self-Consistent Field Input File (example used for band structure calculation):

```
&control
    calculation = 'nscf'
    restart_mode='from_scratch'
    prefix = 'anatase'
    outdir = './outdir'
    pseudo_dir = '-'
/
&system
  ibrav = 0
  nat = 12
  ntyp = 2
  noncolin = .false.
  lspinorb = .false.
  ecutwfc = 220
  occupations = 'fixed'
  smearing = 'gaussian'
  degauss = 0.002
  nspin  = 1
  tot_charge  = 0.0
  nosym = .true.
  noinv = .true.
    nbnd=200
    occupations  = 'tetrahedra'
/
&electrons
    conv_thr=1.1d-8
/
ATOMIC_SPECIES
 Ti  47.86700  ti.fhi.UPF
 O   15.99940  08-o.lda.fhi.UPF
CELL_PARAMETERS cubic
   7.052884613    0.000000000  0.0000000000
   0.000000000    7.052893877  0.0000000000
   0.000000000    0.000000000  17.813258477
```

```
ATOMIC_POSITIONS crystal
Ti     0.9989304300     0.9990218340     0.9889160540
Ti     0.4989346150     0.4990157270     0.4889157940
Ti     0.9989331860     0.4990225240     0.2389181510
Ti     0.4989289910     0.9990164210     0.7389132230
O     0.9989302620     0.9990225790     0.1969983610
O     0.4989269390     0.4990168940     0.6969918920
O     0.9989346180     0.4990128120     0.4469955950
O     0.4989312380     0.9990212260     0.9469954940
O     0.4989334320     0.4990235570     0.2808392350
O     0.9989301330     0.9990146590     0.7808332590
O     0.4989345910     0.9990164110     0.5308359080
O     0.9989308730     0.4990219780     0.0308359370

K_POINTS (automatic)
  8 8 8 0 0 0
```

Variable-Cell Relaxation Input File:

```
&control
    calculation = 'vc-relax'
    prefix = 'anatase'
    outdir = './outdir'
    pseudo_dir = './'
    wfcdir = 'undefined'
    tstress = .false.
    tprnfor = .false.
    wf_collect = .true.
/
&SYSTEM
  ibrav = 0
  nat = 12
  ntyp = 2
  noncolin = .false.
  lspinorb = .false.
  ecutwfc = 220
  occupations = 'fixed'
  smearing = 'gaussian'
  degauss = 0.002
  nspin  = 1
  tot_charge  = 0.0
  nosym = .true.
  noinv = .true.
  nbnd = 248
/
&electrons
    conv_thr=1.1d-8
```

```
    mixing_beta = 0.3
    electron_maxstep = 250
    startingwfc = 'atomic+random'
    diagonalization = 'david'
/
&ions
/
&cell
  cell_dofree = 'ibrav'
/
ATOMIC_SPECIES
 Ti   47.8670   ti.fhi.UPF
 O   15.9994   08-o.lda.fhi.UPF
CELL_PARAMETERS cubic
  7.05288461300000        0.000000000000000E+000  0.000000000000000E+000
  0.000000000000000E+000  7.05289387700000        0.000000000000000E+000
  0.000000000000000E+000  0.000000000000000E+000  17.8132584770000
ATOMIC_POSITIONS crystal
Ti     0.9989304300     0.9990218340     0.9889160540 0 0 0
Ti     0.4989346150     0.4990157270     0.4889157940 0 0 0
Ti     0.9989331860     0.4990225240     0.2389181510 0 0 0
Ti     0.4989289910     0.9990164210     0.7389132230 0 0 0
O     0.9989302620     0.9990225790     0.1969983610 0 0 0
O     0.4989269390     0.4990168940     0.6969918920 0 0 0
O     0.9989346180     0.4990128120     0.4469955950 0 0 0
O     0.4989312380     0.9990212260     0.9469954940 0 0 0
O     0.4989334320     0.4990235570     0.2808392350 0 0 0
O     0.9989301330     0.9990146590     0.7808332590 0 0 0
O     0.4989345910     0.9990164110     0.5308359080 0 0 0
O     0.9989308730     0.4990219780     0.0308359370 0 0 0
K_POINTS automatic
8 8 8 0 0 0
```

Projected Density of States File (Run After SCF and NSCF):

```
&PROJWFC
  prefix= 'anatase',
  outdir= './outdir',
  filpdos= ' anatase_pdos.dat'
/
```

Band Structure Files:

--- Bands Calculation ---

```
&control
    calculation = 'bands'
    restart_mode='from_scratch'
```

```
    prefix = 'anatase'
    outdir = './outdir'
    pseudo_dir = '-'
/
&system
  ibrav = 0
  nat = 12
  ntyp = 2
  noncolin = .false.
  lspinorb = .false.
  ecutwfc = 220
  occupations = 'fixed'
  smearing = 'gaussian'
  degauss = 0.002
  nspin  = 1
  tot_charge  = 0.0
  nosym = .true.
  noinv = .true.
    nbnd=200
/
&electrons
    conv_thr=1.1d-8
/
ATOMIC_SPECIES
 Ti  47.86700  ti.fhi.UPF
 O   15.99940  08-o.lda.fhi.UPF
CELL_PARAMETERS cubic
   7.052884613    0.000000000  0.0000000000
   0.000000000    7.052893877  0.0000000000
   0.000000000    0.000000000  17.813258477
ATOMIC_POSITIONS crystal
Ti     0.9989304300     0.9990218340     0.9889160540
Ti     0.4989346150     0.4990157270     0.4889157940
Ti     0.9989331860     0.4990225240     0.2389181510
Ti     0.4989289910     0.9990164210     0.7389132230
O     0.9989302620     0.9990225790     0.1969983610
O     0.4989269390     0.4990168940     0.6969918920
O     0.9989346180     0.4990128120     0.4469955950
O     0.4989312380     0.9990212260     0.9469954940
O     0.4989334320     0.4990235570     0.2808392350
O     0.9989301330     0.9990146590     0.7808332590
O     0.4989345910     0.9990164110     0.5308359080
O     0.9989308730     0.4990219780     0.0308359370


K_POINTS {crystal_b}
```

```
8
  0.0 0.0 0.0 10 !Gamma
  0.5 0.0 0.0 10 !X
  0.5 0.0 0.5 10 !R
  0.0 0.0 0.5 10 !Z
  0.0 0.0 0.0 10 !Gamma
  0.5 0.5 0.0 10 !M
  0.5 0.5 0.5 10 !A
  0.0 0.0 0.5 10 !Z
```

--- Preparing Output for ‘plotband’ ---

```
&bands
  outdir='./outdir/'
  prefix='anatase'
  filband='anatase.bands.dat'
/
```

--- Plotting the Bands, ‘plotband’ ---

```
anatase.bands.dat
2 18
anatase.bands.xmgr
anatase.bands.ps
7.4320
2 7.4320
```

Appendix B. Example OCEAN 2.5.2 Input File:

```
ppdir '../'
dft{qe}
para_prefix{ mpirun -np 56 }

##############################

nkpt { 16 16 12 }
ngkpt { 16 16 12 }

screen.nkpt { 2 2 2 }
screen.nbands 200

nbands 248

mixing { 0.3 }

acell { 7.052884613  7.052893877  17.813258477 }
rprim {
    1.0 0.0 0.0
    0.0 1.0 0.0
    0.0 0.0 1.0}

ntypat 2
znucl { 22 8 }
zsymb { Ti O}

ppdir { '../' }
pp_list{ ti.fhi
        08-o.lda.fhi }

natom 12
typat { 1 1 1 1 2 2 2 2 2 2 2 2 }

xred {
  0.998930430   0.999021834   0.988916054
  0.498934615   0.499015727   0.488915794
  0.998933186   0.499022524   0.238918151
  0.498928991   0.999016421   0.738913223
  0.998930262   0.999022579   0.196998361
  0.498926939   0.499016894   0.696991892
  0.998934618   0.499012812   0.446995595
  0.498931238   0.999021226   0.946995494
  0.498933432   0.499023557   0.280839235
  0.998930133   0.999014659   0.780833259
  0.498934591   0.999016411   0.530835908
```

```
  0.998930873   0.499021978   0.030835937}

ecut 350
toldfe 1.1d-8
tolwfr 1.1d-16

nstep 250

# Static dielectric const https://aip.scitation.org/doi/pdf/10.1063/1.435102
diemac 5.62

CNBSE.xmesh { 8 8 8 }

opf.fill{ 22 ti.fill }
opf.opts{ 22 ti.opts }

# edge information # number of edges to calculate # atom number, n quantum number, l quantum
number
edges{ -22 2 1 }

cnbse.broaden{ 0.1 }

screen.shells{ 4.0 }
cnbse.rad{ 4.0 }

spin-orbit 3.828

scfac 0.8
occopt 1
core_offset .true.
bshift{48} #Sets the number of valence bands in the modified code
```
